# Mena/VASP and αII-Spectrin complexes regulate cytoplasmic actin networks in cardiomyocytes and protect from conduction abnormalities and dilated cardiomyopathy

**DOI:** 10.1186/1478-811X-11-56

**Published:** 2013-08-12

**Authors:** Peter M Benz, Carla J Merkel, Kristin Offner, Marco Abeßer, Melanie Ullrich, Tobias Fischer, Barbara Bayer, Helga Wagner, Stepan Gambaryan, Jeanine A Ursitti, Ibrahim M Adham, Wolfgang A Linke, Stephan M Feller, Ingrid Fleming, Thomas Renné, Stefan Frantz, Andreas Unger, Kai Schuh

**Affiliations:** 1 Institute of Physiology I, University of Würzburg, D-97070 Würzburg, Germany; 2 Institute for Vascular Signalling, Goethe-University Frankfurt, D-60590 Frankfurt, Germany; 3 Department of Neurology, University of Bonn, D-53105 Bonn, Germany; 4 Comprehensive Heart Failure Center, University of Würzburg, D-97070 Würzburg, Germany; 5 Institute of Clinical Biochemistry and Pathobiochemistry, University of Würzburg, D-97070 Würzburg, Germany; 6 Sechenov Institute of Evolutionary Physiology and Biochemistry, Russian Academy of Sciences, St. Petersburg, Russia; 7 Department of Physiology, University of Maryland School of Medicine, Baltimore, MD 21201 USA; 8 Institute of Human Genetics, University of Göttingen, D-37073 Göttingen, Germany; 9 Department of Cardiovascular Physiology, Ruhr University Bochum, D-44780 Bochum, Germany; 10 Biological Systems Architecture Group, University of Oxford, Oxford OX3 9DS UK; 11 Section Tumor Biology, Institute of Molecular Medicine, ZAMED, Martin-Luther-University Halle-Wittenberg, D-06120 Halle (Saale), Germany; 12 Department of Molecular Medicine and Surgery and Center for Molecular Medicine, Karolinska Institutet, 17176 Stockholm, Sweden; 13 Institute for Clinical Chemistry, University Hospital Eppendorf, D-20246 Hamburg, Germany

**Keywords:** Actin, Heart, Mena/VASP, Spectrin, Dilated cardiomyopathy

## Abstract

**Background:**

In the heart, cytoplasmic actin networks are thought to have important roles in mechanical support, myofibrillogenesis, and ion channel function. However, subcellular localization of cytoplasmic actin isoforms and proteins involved in the modulation of the cytoplasmic actin networks are elusive. Mena and VASP are important regulators of actin dynamics. Due to the lethal phenotype of mice with combined deficiency in Mena and VASP, however, distinct cardiac roles of the proteins remain speculative. In the present study, we analyzed the physiological functions of Mena and VASP in the heart and also investigated the role of the proteins in the organization of cytoplasmic actin networks.

**Results:**

We generated a mouse model, which simultaneously lacks Mena and VASP in the heart. Mena/VASP double-deficiency induced dilated cardiomyopathy and conduction abnormalities. In wild-type mice, Mena and VASP specifically interacted with a distinct αII-Spectrin splice variant (SH3*i*), which is in cardiomyocytes exclusively localized at Z- and intercalated discs. At Z- and intercalated discs, Mena and β-actin localized to the edges of the sarcomeres, where the thin filaments are anchored. In Mena/VASP double-deficient mice, β-actin networks were disrupted and the integrity of Z- and intercalated discs was markedly impaired.

**Conclusions:**

Together, our data suggest that Mena, VASP, and αII-Spectrin assemble cardiac multi-protein complexes, which regulate cytoplasmic actin networks. Conversely, Mena/VASP deficiency results in disrupted β-actin assembly, Z- and intercalated disc malformation, and induces dilated cardiomyopathy and conduction abnormalities.

## Background

Actin dynamics regulate a tremendous range of cellular functions. Currently, it is believed that different actin isoforms and their associated actin-binding proteins form multiple specialized actin networks, which accomplish the large diversity of tasks [1]. In the heart, cytoplasmic actin networks are thought to have important roles in mechanical support, myofibrillogenesis, and ion channel function [2] and elevated levels of cytoplasmic actin have been reported in dilated cardiomyopathy (DCM) [3]. Indeed, the α-cardiac actin isoform is the major constituent of thin filaments in the sarcomeres, but circumstantial evidence supports the existence of a different actin isoform at Z- and intercalated discs, possibly β- or γ-cytoplasmic actin [2,4,5].

Proteins of the Enabled/vasodilator-stimulated phosphoprotein (Ena/VASP) family are important mediators in cytoskeleton control, linking cyclic nucleotide signaling pathways to actin assembly [6]. In the mammalian heart, two Ena/VASP proteins are predominantly expressed, mammalian Enabled (Mena) and VASP [7]. Mena and VASP increase actin filament assembly by their intrinsic polymerase and anti-capping activities [8] and the latter are impaired by the cyclic nucleotide-dependent phosphorylation of the proteins [9-11]. Because of their ability to modulate distinct modes of actin organization, it has been proposed that Mena and VASP may serve as higher order regulators of the actin cytoskeleton [12]. Given their essential role in regulating actin dynamics, it was surprising that the deletion of the genes encoding Mena or VASP in mice resulted in only mild neuronal or platelet dysfunction [13-16]. One explanation for the lack of a pronounced phenotype could be the mutual functional compensation of the proteins. Consistent with this hypothesis, functional abnormalities in Mena^−/−^ or VASP^−/−^ mice were detected in tissues that predominantly express only one of the family members [7] and human VASP has been shown to rescue the lethal phenotype in Drosophila Ena^−/−^ mutants [17]. Furthermore, Mena/VASP double-deficient mice die perinatally and suffer from severe defects in brain mor-phology [18]. However, a single allele of Mena is sufficient to rescue the embryonic lethal phenotype [13].

In cardiac muscle cells, scaffold proteins of the Spectrin family coordinate macromolecular protein complexes, which organize transmembrane signaling proteins and at the same time enable muscle cells to resist the extreme forces of contraction [19-21]. αII-Spectrin is abundant at the lateral plasma membrane but it is also enriched at Z- and intercalated discs close to the edges of the sarcomeres [22]. At the intercalated discs, αII-Spectrin is a critical component of the transitional junction, an axial interface located where the thin actin filaments of the terminal sarcomere continue into the adherens junctions [4]. The critical role of αII-Spectrin in maintaining cardiac integrity is demonstrated by the fact that αII-Spectrin-deficient embryos die *in utero* and exhibit abnormal cardiac shape, cardiac dilation, and thinning of the myocardium [23]. These cardiac deformities are the likely cause of the embryonic lethality and reminiscent of the cardiac phenotypes induced by the loss of proteins that regulate actin dynamics [23]. Given that the direct interaction of VASP and αII-Spectrin initiates β-actin filament assembly and stabilizes cell-cell contacts [10], it is tempting to speculate that αII-Spectrin:Mena/VASP complexes may be important for the formation and stability of cytoplasmic actin networks in the heart.

In the present study, we aimed to address cardiac functions of Mena and VASP using a combination of mouse models lacking either Mena or VASP, or both proteins in the heart. Mechanistically, we focused on the role of Mena/VASP in the organization of cytoplasmic actin networks and whether this has implications for mechanical support, myofibrillogenesis, and ion channel function in the mammalian heart.

## Results

### Stage-dependent expression of cytoskeletal proteins in the mouse heart

**Figure 1:**
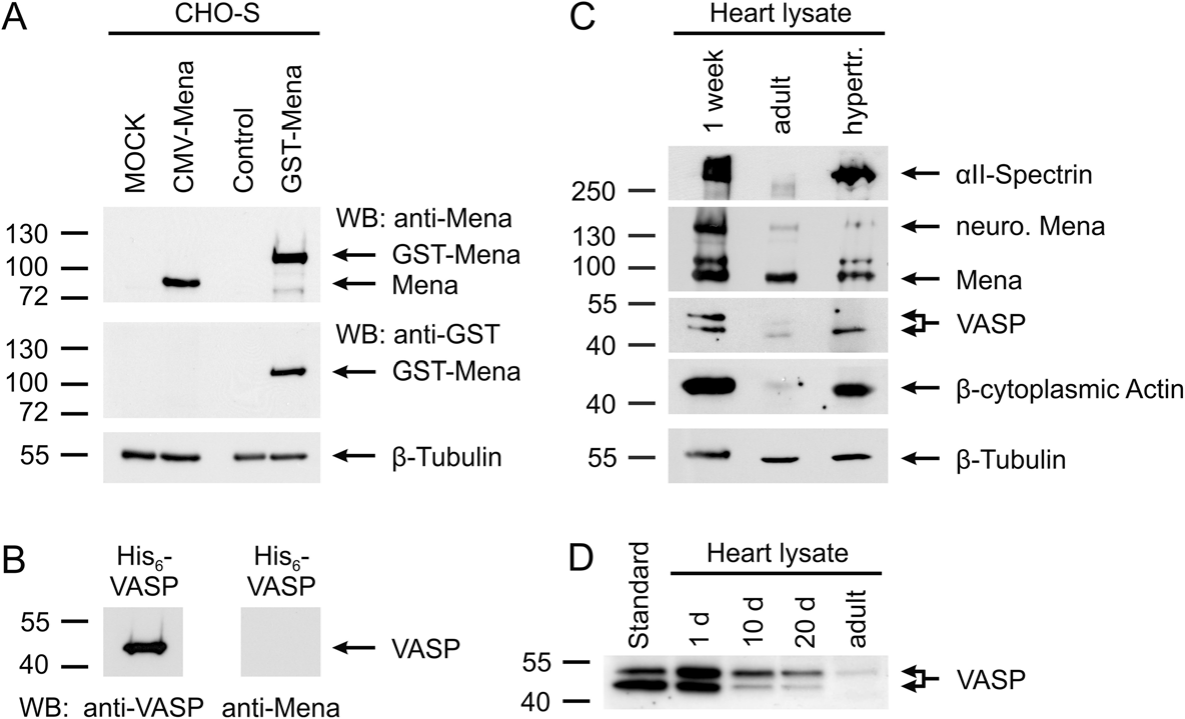
**Stage-dependent expression of cytoskeletal proteins in the mouse heart. (A, B)** Characterization of Mena-specific antibodies. **(A)** CHO-S cells, either transiently transfected with murine Mena (CMV-Mena) or stably transfected with GST-tagged Mena (GST-Mena), were lysed and analyzed by Western blotting with Mena-specific (upper panel) or GST-specific (middle panel) antibodies. As control, lysates of MOCK-transfected cells or lysates of CHO-S cells without stable integration of GST-Mena were run on the same gels. β-Tubulin served as loading control (lower panel). **(B)** 100 ng purified VASP protein was probed by Western blotting with anti-VASP (left panel) and anti-Mena (right panel) antibodies. **(C**, **D)** Stage-dependent expression of cytoskeletal proteins in the mouse heart. **(C)** Lysates of 1-week-old, adult, and hypertrophic adult mouse hearts were probed by Western blotting with antibodies against αII-Spectrin, Mena, VASP, and β-cytoplasmic actin. Blotting for β-Tubulin was used as invariant loading control. Two Mena isoforms are expressed in the mouse heart, the general (~80 kDa) and the neuronal-specific (~140 kDa) form. Depending on its phosphorylation state, VASP migrates at 46 or 50 kDa. **(D)** VASP expression in the mouse heart at postnatal day 1, 10, and 20 and in the adult heart.

To determine the cardiac functions of Mena and VASP and the associated cytoskeletal proteins, αII-Spectrin and actin, we analyzed the protein levels in wild-type mouse hearts by Western blotting. For this, we generated a polyclonal anti-Mena antibody using a fusion protein that comprises the LERER region of mouse Mena for immunization and the specificity of the purified antibodies was controlled by Western blotting. Consistent with the reported apparent molecular weight of mouse Mena in Western blots [7], our antibody detected a protein band at ~80 kDa, which was only seen in lysates of Mena-transfected, but not in the MOCK-transfected cells (Figure 1A, upper panel, lanes 1 and 2). We also investigated lysates of CHO-S cells with a stable integration of GST-Mena [24]. Our Mena-specific antibody (Figure 1A, upper panel, lanes 3 and 4) as well as a GST-tag-specific antibody (middle panel, lanes 3 and 4) displayed a signal at ~110 kDa, which was only visible in lysates of GST-Mena expressing cells but not in controls. Consistent with the absence of LERER repeats in VASP [6], our Mena antibody did not detect the purified VASP protein in Western blots (Figure 1B).

In the mouse heart, at least two Mena isoforms exist. The ubiquitously expressed isoform, which migrates with an apparent molecular weight of ~80 kDa and a splice variant, which is predominantly expressed in neuronal tissue and migrates at about 140 kDa (Figure 1C). VASP migrates with an apparent molecular weight of 46 kDa in SDS-PAGE, but PKA-mediated phosphorylation of VASP on Ser157 induces an electrophoretic mobility shift of the protein from 46 to 50 kDa (Figures 1C and D). Thus, depending on PKA activity in the heart, one or two protein bands can be observed in Western blots. We investigated protein expression in hearts of 1-week-old and adult mice under basal conditions, as well as the hearts of adult mice subjected to transverse aortic constriction (TAC) for 3 weeks to induce left-ventricular hypertrophy. The expression patterns of the investigated proteins showed a comparable trend. αII-Spectrin, Mena, VASP, and β-cytoplasmic actin were highly expressed in 1-week old hearts and protein levels declined until adulthood (Figures 1C and D). Consistent with the reactivation of the fetal gene expression program, protein levels were significantly increased in hypertrophic hearts (Figure 1C). Taken together, the expression of Mena and VASP paralleled the expression of β-cytoplasmic actin and αII-Spectrin in the 1-week old, adult, and hypertrophied mouse heart.

### Targeted disruption of mouse Mena

**Figure 2:**
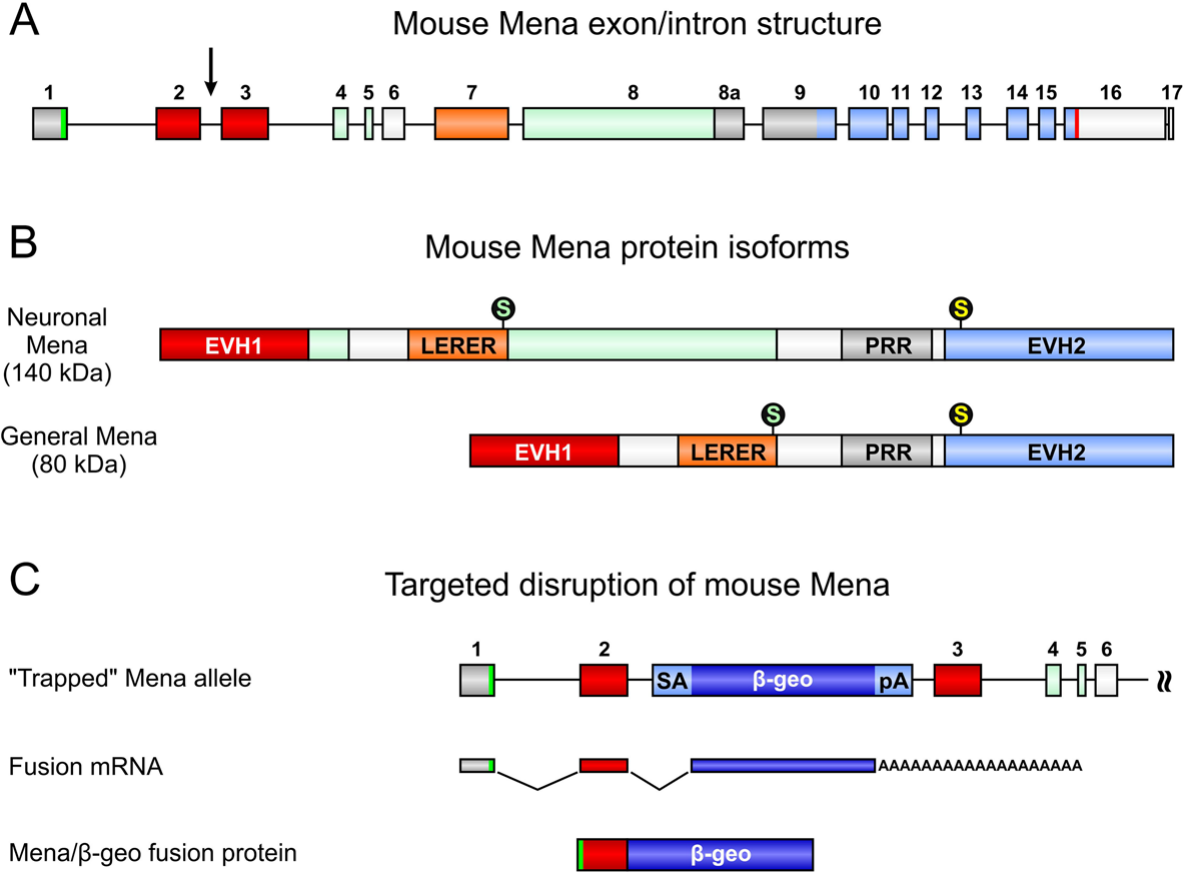
**Targeted disruption of the mouse Mena gene. (A)** The mouse Mena gene is located on chromosome 1 spanning about 115 kb of genomic sequence. The translational start and termination codons are given in green (exon 1) and red (exon 16), respectively. The neuronal-specific exons are shown in green. Exons 2 and 3, encoding the Ena/VASP homology 1 (EVH1) domain are shown in red. Exons encoding the LERER repeats, the proline-rich region (PRR), and the EVH2 domain are indicated in orange, grey, and light blue, respectively. The insertion of a gene trap vector in intron 2 of the Mena gene is indicated by a black arrow **(**compare **C)**. **(B)** Depending on differential splicing, a neuronal-specific and a general Mena protein isoform exist. Mena is phosphorylated at serine 236 by PKA (green “S”) and at serine 376 by PKG (yellow “S”; numbering according to the 541aa general Mena isoform). **(C)** In the ES cell clone RRG138, the gene trap vector is inserted in Mena intron 2. The gene trap vector consists of a splice acceptor side (SA), a polyadenylation signal (pA), and β-geo, which encodes β-galactosidase and neomycin resistance [26]. Insertion of the gene trap vector disrupts the Mena gene and results in the expression of a Mena/β-geo fusion protein. Because critical parts of the N-terminal EVH1 domain are missing in the fusion protein, Mena/β-geo most likely lacks all known endogenous Mena functions.

The mouse Mena gene is composed of 17 exons located on chromosome 1 (Figure 2A) and a neuronal-specific and a ubiquitous Mena protein isoform are generated by differential splicing. Both isoforms share the N-terminal Ena/VASP homology 1 (EVH1) domain, the LERER repeats, the proline-rich region (PRR), and the C-terminal EVH2 domain (Figure 2B and [25]). To target Mena gene expression, we used the murine embryonic stem (ES) cell line RRG138, in which one Mena allele has been disrupted by insertion of a gene trap vector in intron 2 (Figure 2A, black arrow and 2C). Upon transcription/translation of the “trapped” Mena gene, the endogenous full-length protein is replaced by a fusion-protein encoded by the first two Mena exons and the reporter gene. Because Mena exon 3 encodes critical parts of the N-terminal EVH1 domain, a key feature of all known Mena protein isoforms, the resulting Mena/β-geo fusion protein most likely lacks all endogenous Mena functions (Figure 2C). Following blastocyst injection of the ES cells, mice that were homozygous for the gene-trap insertion (Mena^GT/GT^) were generated. Mena^GT/GT^ mice were viable, fertile, and macroscopically indistinguishable from wild-type controls.

### Generation of Mena/VASP double-deficient mice (Mena^GT/GT^VASP^−/−^, dKO)

**Figure 3:**
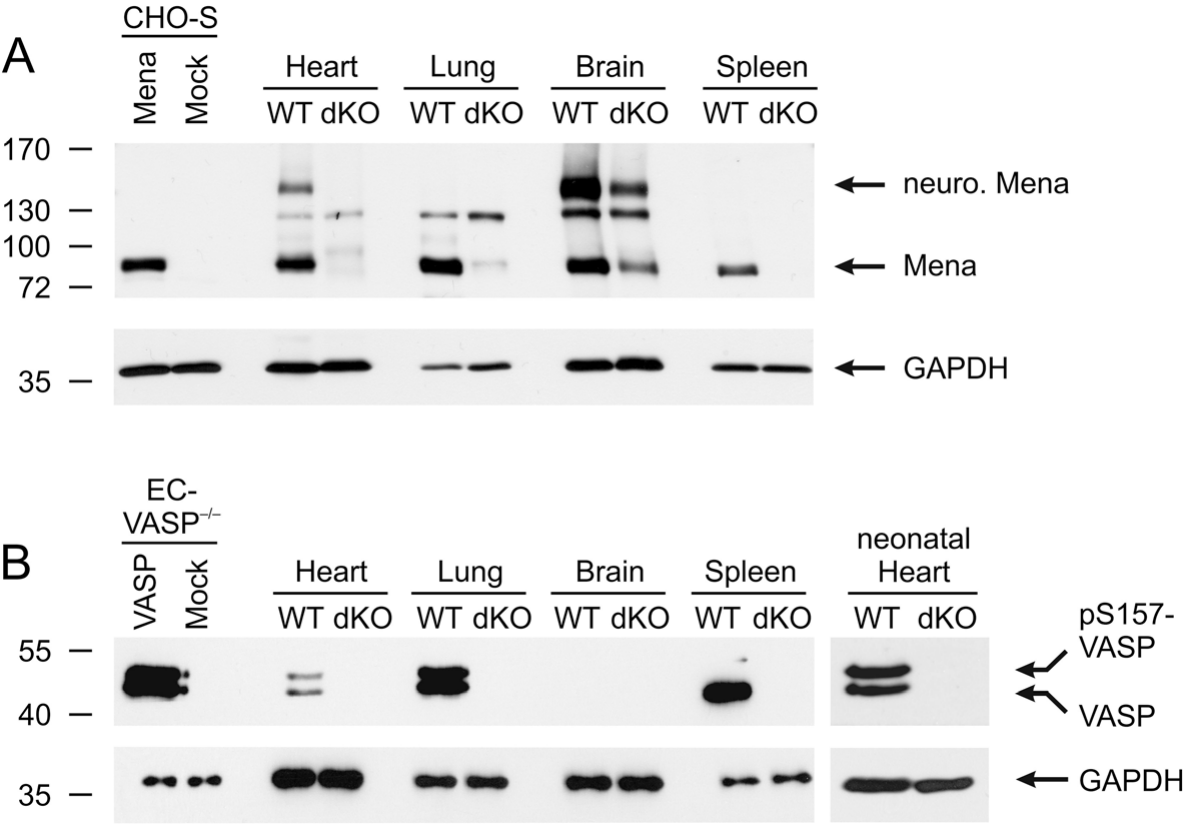
**Mena/VASP expression in wild-type and Mena**^**GT/GT**^**VASP**^**−/− **^**mouse organs.** Western blots to analyze Mena **(A)** and VASP **(B)** protein levels in heart, lung, brain, and spleen of wild-type (WT) and Mena^GT/GT^VASP^−/−^ (dKO) mice. GAPDH and lysates of Mena- or VASP-transfected cells were used as controls. **(A)** The general Mena protein isoform (~80 kDa) is present in all analyzed organs, the neuronal splice variant (~140 kDa) is only detected in heart and brain. Whereas Mena protein in dKO brain is moderately reduced to ~30% of wild-type levels, Mena is almost undetectable in heart, lung, and spleen. The 130 kDa band seen in heart, lung, and brain likely represents a cross-reaction with an unrelated protein. **(B)** VASP (46 or 50 kDa, depending on phosphorylation state) expression is high in the neonatal heart and the adult lung and spleen, low in the adult heart, and nearly undetectable in the brain. No VASP protein is detected in dKO organs.

When we analyzed Mena protein levels in our Mena^GT/GT^ mice, we found that specifically in the brain, but not in other organs, the gene trap was “leaky” [27,28], resulting in a low amount of endogenous full length protein (data not shown but compare Mena protein levels in the Mena^GT/GT^VASP^−/−^ organs, Figure 3A, dKO lanes). We speculated that the residual Mena expression in the brain of Mena^GT/GT^ mice may overcome the embryonic lethal phenotype of Mena/VASP double-deficient mice and crossed the Mena^GT/GT^ animals with our VASP^−/−^ mice [16]. Indeed, the remaining Mena protein expression in the brain was sufficient to produce viable mice (Mena^GT/GT^VASP^−/−^). In agreement with our previous studies [7], the general Mena isoform (80 kDa) was detected in protein extracts from wild-type heart, lung, brain, and spleen. In wild-type heart and brain the antibodies also detected the neuronal splice variant (140 kDa; Figure 3A, WT lanes). In lysates from Mena^GT/GT^VASP^−/−^ mice, Mena expression was nearly undetectable in heart, lung, and spleen (Figure 3A, dKO lanes). In contrast, in brain protein extracts from Mena^GT/GT^VASP^−/−^ mice, both Mena isoforms were only moderately decreased (by approximately 70%) versus protein levels in wild-type mice (Figure 3A). We could not detect VASP protein expression in any organ from Mena^GT/GT^VASP^−/−^ mice (Figure 3B).

### Mena/VASP double-deficiency induces cardiac fibrosis and hypertrophy

**Figure 4:**
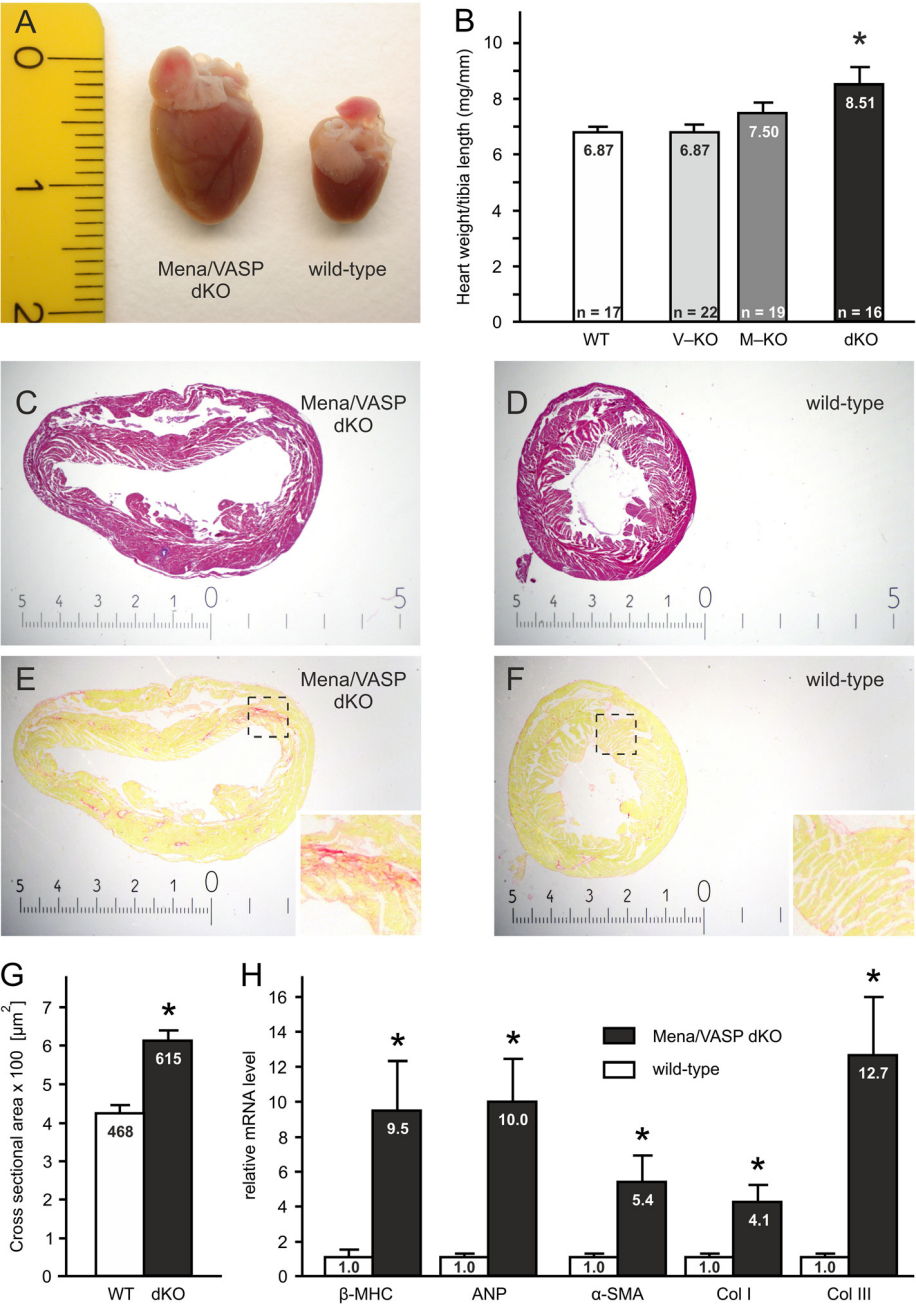
**Enlarged and dilated hearts in Mena**^**GT/GT**^**VASP**^**−/− **^**mice**. Mena^GT/GT^VASP^−/−^ (dKO) animals display macroscopically enlarged hearts (**A**, photography, scale in cm) and significantly increased heart weight to tibia length ratios as compared to wild-type (WT) controls (**B**; ANOVA, *P<0.05). **(C**, **D)** Hematoxilin and eosin stained cross sections of Mena^GT/GT^VASP^−/−^**(C)** and wild-type **(D)** hearts, demonstrating dilated ventricles in the double-deficient animals. **(E**, **F)** Picrosirius red stained heart cross sections of dKO **(E)** and WT **(F)** mice to visualize collagen fibers/interstitial fibrosis. Magnified views of the indicated areas are shown as insets. The scales in images **C**-**F** are given in mm. **(G)** Cross sectional areas of cardiomyocytes from WT and Mena^GT/GT^VASP^−/−^ hearts quantified by morphometric analyses (five animals per group, 25 cardiomyocytes per animal, *P<0.05). **(H)** Quantitative real-time RT-PCR revealed increased mRNA levels of cardiac hypertrophy markers (β-myosin heavy chain, β-MHC; atrial natriuretic peptide, ANP) and fibrosis markers (α-smooth muscle actin, α-SMA; collagen I, Col I; and collagen III, Col III; *P<0.05; n=6) in dKO mice vs. WT controls.

We first investigated heart weight to tibia length (HW/TL) ratios in adult VASP-deficient (V-KO), Mena^GT/GT^ (M-KO), and Mena^GT/GT^VASP^−/−^ (dKO) animals. The HW/TL ratio of VASP-deficient mice was undistinguishable from wild-type controls and although the ratio of the Mena^GT/GT^ mice was slightly elevated, this trend did also not reach significance. In contrast to the individual knockouts, the HW/TL ratio of the Mena^GT/GT^VASP^−/−^ animals was significantly increased by over 20% compared to wild-type controls (Figure 4A and B).

**Figure 5:**
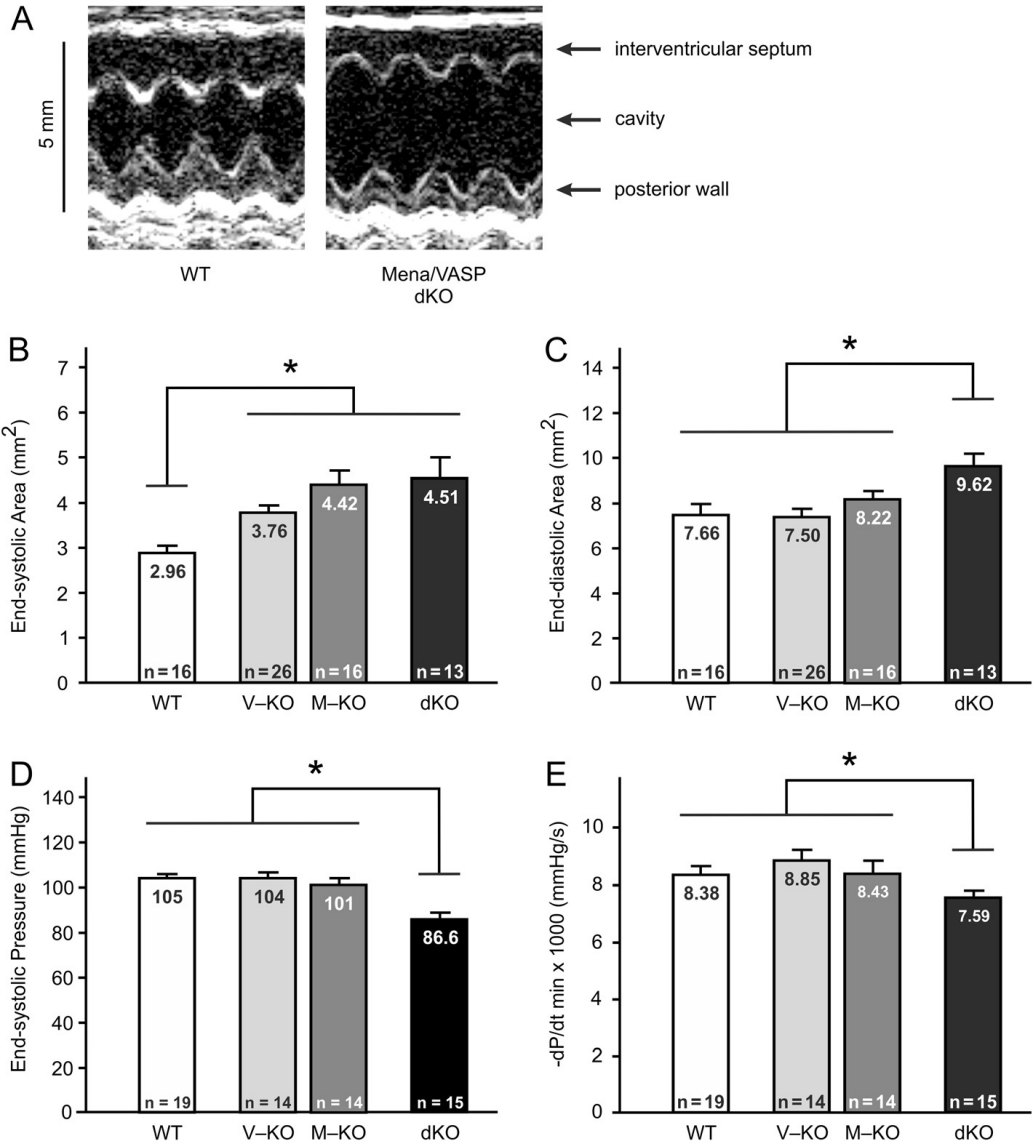
**Mena/VASP double-deficiency induces dilated cardiomyopathy. (A-C)** Echocardiography. **(A)** Representative M-mode recordings at the mid-papillary muscle level of wild-type (WT) and Mena^GT/GT^VASP^−/−^ (dKO) mice. **(B**, **C)** Statistical analyses of the echocardiographic measurements demonstrated a significantly increased left intraventricular end-systolic area in VASP^−/−^ (V-KO), Mena^GT/GT^ (M-KO), and Mena^GT/GT^VASP^−/−^ (dKO) animals as compared to wild-type controls **(B)**. The left intraventricular end-*diastolic* area was only significantly increased in the dKO animals **(C)**. **(D**, **E)** Left ventricular catheterization revealed a significantly reduced end-systolic pressure **(D)** and a significantly reduced rate of pressure decline **(E)** in the left ventricle of dKO mice. Bars represent the mean ± S.E.M (ANOVA, *P<0.05).

Histological analyses of Mena^GT/GT^VASP^−/−^ hearts revealed substantial dilation of both ventricles compared to wild-type hearts (Figure 4C and D), indicating eccentric hypertrophy. Dilated ventricles were not observed in the Mena^GT/GT^ or VASP^−/−^ mice (compare end-diastolic areas in echocardiography, Figure 5C). In the Mena^GT/GT^VASP^−/−^ mice, cardiac dilation was also associated with marked interstitial fibrosis (Figure 4E and F).

Consistent with cardiac fibrosis and hypertrophy, cross sectional areas of cardiomyocytes were significantly elevated in the Mena^GT/GT^VASP^−/−^ animals (Figure 4G) as were mRNA levels of the cardiac hypertrophy markers; β-myosin heavy chain and atrial natriuretic peptide, and the fibrosis markers; α-smooth muscle actin, collagen I and collagen III (Figure 4H).

### Mena/VASP double-deficiency induces DCM and conduction abnormalities

Decompensated eccentric hypertrophy and cardiac dilation are characterized by a reduced functional performance of the heart [29]. Therefore, we investigated left ventricular function by echocardiography under basal conditions. M-mode and two-dimensional recordings at the mid-papillary muscle level revealed clear differences between wild-type and mutant mice. The left ventricles of Mena^GT/GT^, VASP^−/−^ and Mena^GT/GT^VASP^−/−^ animals displayed significantly increased end-systolic areas compared to wild-type controls (Figure 5A and B). However, the Mena^GT/GT^ and Mena^GT/GT^VASP^−/−^ animals were more affected than the VASP^−/−^ mice. A significant increase in the end-*diastolic* area was only observed in the Mena^GT/GT^VASP^−/−^ mice (Figure 5A and C) and is in agreement with the histological data (see Figure 4). Consistent with an impaired cardiac contraction, Mena^GT/GT^VASP^−/−^ mice displayed a significantly reduced fractional shortening compared to wild-type animals (WT 61.4% vs. dKO 53.0%, *P<0.05, n=13). To directly assess hemodynamic parameters in wild-type and mutant animals, we analyzed left ventricular pressures by invasive catheterization. Consistent with the occurrence of dilated ventricles, the end-systolic pressure was significantly reduced in the Mena^GT/GT^VASP^−/−^ but not in the wild-type, Mena^GT/GT^, or VASP^−/−^ animals (Figure 5D). Rates of left ventricular pressure increase were not significantly different between the groups (data not shown). Consistent with the pronounced interstitial fibrosis, however, the rate of left ventricular pressure decline was diminished in Mena^GT/GT^VASP^−/−^ mice (Figure 5E).

**Figure 6:**
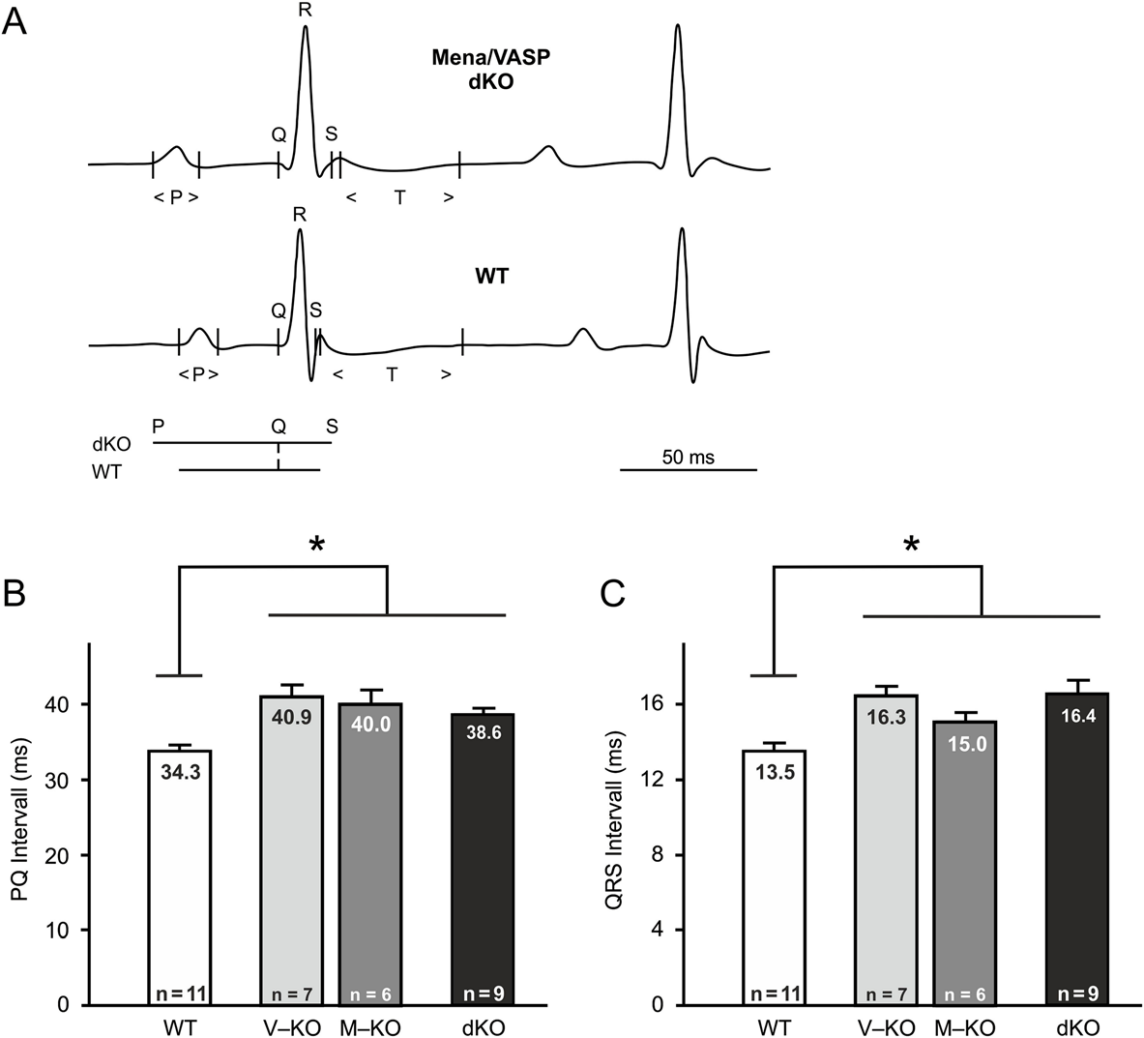
**Mena/VASP double-deficiency results in conduction abnormalities.** Electrocardiography (ECG). **(A)** Original, high resolution lead I ECG recording of a representative Mena^GT/GT^VASP^−/−^ (dKO, upper panel) and wild-type (WT, lower panel) mouse. Q, R, and S spikes and the P- and T-waves are indicated. A comparison of the PQ and QRS intervals from dKO and WT mice is shown below the ECG recording of the WT. **(B**, **C)** Statistical analysis of the ECG recordings demonstrated significantly prolonged PQ **(B)** and QRS **(C)** intervals in VASP^−/−^ (V-KO), Mena^GT/GT^ (M-KO), and Mena^GT/GT^VASP^−/−^ (dKO) mice. Bars represent the mean ± S.E.M, one-way analysis of variance (ANOVA), *P<0.05).

Next, we investigated the impact of Mena/VASP deficiency on the electrical conduction in the mammalian heart and performed high resolution ECG recordings (Figure 6). Although heart rates were nearly identical in all strains (wild-type 441 bpm, VASP^−/−^ 434, Mena^GT/GT^ 432 bpm, Mena^GT/GT^VASP^−/−^ 439 bpm), PQ and QRS intervals, which reflect the intra-atrial and the intra-ventricular propagation of electrical signals, respectively, were significantly prolonged in the Mena^GT/GT^, VASP^−/−^, and Mena^GT/GT^VASP^−/−^ mice. Interestingly, the differences in the Mena^GT/GT^VASP^−/−^ animals were statistically indistinguishable from those observed in the Mena^GT/GT^ and VASP^−/−^ mice (Figure 6).

### Mena is localized at Z- and intercalated discs of mouse cardiomyocytes

**Figure 7:**
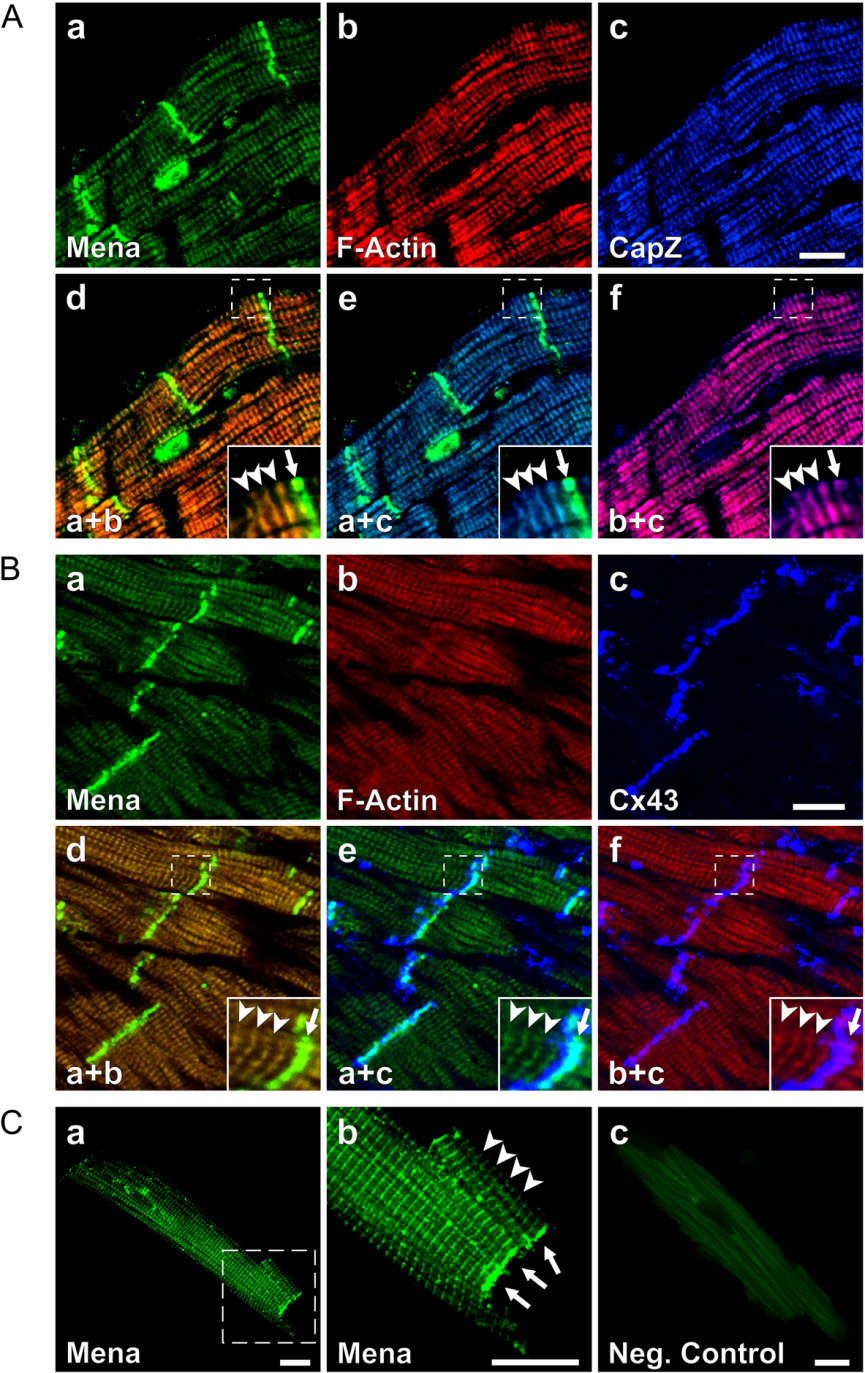
**Mena is localized at Z- and intercalated discs of mouse cardiomyocytes. (A, B)** Confocal microscopy images of adult mouse heart cryosections stained for Mena (**a**, green), non-isoform selective F-actin (**b**,red), and CapZ (**A**, image **c**, blue) or Connexin 43 (Cx43, **B**, image **c**, blue). Mena and F-actin colocalized with CapZ at Z-dics (**A**, merged images **d**-**f**, arrowheads) and with Cx43 at intercalated discs (**B**, merged images **d**-**f**, arrows). **(C)** Adult mouse cardiomyocytes were single-stained with Mena-specific antibodies followed by fluorescence-conjugated secondary antibodies (images **a** and b). Image **b** shows a magnified view of the area indicated in image **a**. Mena localized to Z-discs (arrowheads) and was strongly enriched at the cell termini (arrows). Using identical laser intensities and capture settings, the fluorescent secondary antibodies alone did not reveal any cross-striated signals (image **c**, negative control). Scale bars 15 μm.

Circumstantial evidence suggests that cytoplasmic actin networks are present at Z- and intercalated discs of cardiomyocytes with implications for mechanical support, myofibrillogenesis and ion channel function [1,2,4,5]. To determine whether or not the regulation of cytoplasmic actin dynamics at Z- and intercalated discs may be exerted through Mena and VASP, we investigated the sub-cellular distribution of the proteins by confocal microscopy and initially focused on Mena, which is more robustly expressed in the adult heart. In adult wild-type hearts, a fraction of Mena was localized at Z-discs, where it colocalized with CapZ, a protein that acts as a barbed end capping protein for F-actin and that is found at the end of the thin filaments in the Z-disc (Figure 7A). The majority of Mena, however, was detected at intercalated discs, where it at least partially colocalized with Connexin 43, a critical component of the gap junctions, which carry the electrical stimulus from cell to cell (Cx43, Figure 7B). To exclude channel crosstalk and sectioning artifacts, we also analyzed the subcellular distribution of Mena in isolated murine cardiac myocytes. Consistent with the findings from the intact heart, Mena was localized to Z-discs of isolated cardiomyocytes as well as to cell termini, which correspond to the intercalated discs in heart tissue (Figure 7C).

**Figure 8:**
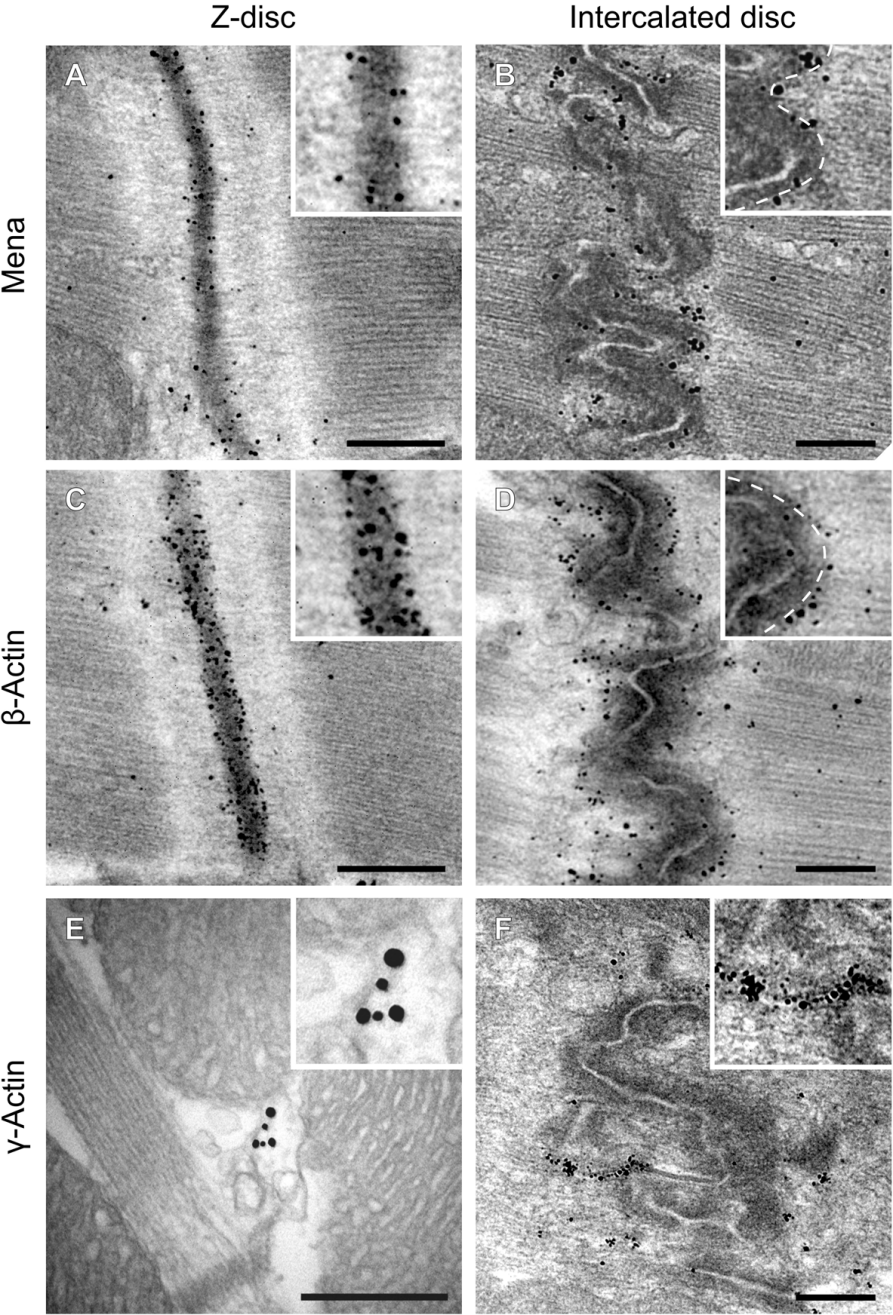
**Mena and β-actin localize to the edges of sarcomeres at Z- and intercalated discs.** Immuno-electron microscopy of Z- and intercalated disc regions in mouse papillary muscle. Sections were labelled with antibodies to Mena **(A**, **B)**, β-actin **(C**, **D)**, and γ-actin **(E**, **F)**. The dashed line in the insets of **B** and **D** indicates the cytoplasmic edge of the dark plaque material that coats the plasma membranes where the thin filaments of the terminal sarcomeres lead into the adherens junctions. **(E)** Most γ-Actin immunogold labeling was observed at filamentous structures, which project from the upper and lower terminals of Z-discs towards the mitochondria. Scale bars 500 nm.

Conventional confocal microscopy has a limited axial resolution of >200 nm. Thus it is difficult to distinguish whether the proteins detected are truly Z-disc proteins or simply associated with nearby structures, i.e. T-tubules. To more accurately determine the subcellular distribution of Mena, we subjected ultrathin sections of murine hearts to electron microscopy (Figure 8A and B). Consistent with the results of the confocal microscopy studies, a fraction of Mena immunoreactivity was detected at Z-discs and the gold particles especially localized towards the edges of the adjacent sarcomeres (Figure 8A). The strongest Mena immunogold signal, however, was observed at the intercalated disc while the plasma membrane itself was not labeled. Indeed the majority of gold particles accumulated within the cytoplasmic edge of the dark plaque material that coats the membranes where the thin filaments of the terminal sarcomeres lead into the adherens junctions (Figure 8B). We also investigated the subcellular distribution of cytoplasmic actin isoforms. Similar to Mena, β-cytoplasmic actin was also detected at Z- and intercalated discs. At Z-discs, however, β-actin labeling was not limited to the edges of sarcomeres, but instead was almost evenly distributed, indicating that β-actin is an integral component of the Z-discs. At intercalated discs, again most gold particles localized to the edges of the adjacent sarcomeres and closely matched the sub-cellular distribution of Mena (Figure 8C and D). In contrast to Mena and β-actin, γ-actin was neither detectable at the Z-discs nor within the I-bands of the sarcomeres. Instead, most gold particles were observed at the mitochondria in the vicinity of I-Z-I bands in cardiac myofibrils. High power magnifications suggested that γ-actin is an integral part of the filamentous structures, which project from the upper and lower terminals of Z-discs towards the mitochondria (Figure 8E). Weak immunoreactivity with small gold particle clusters was also detected inside the intercalated discs, likely representing γ-actin association with local microcompartments. In contrast to Mena and β-actin, however, no γ-actin labeling was detected at the edges of the terminal sarcomeres (Figure 8F).

### Mena and VASP specifically interact with SH3*i*, an αII-Spectrin splice variant

**Figure 9:**
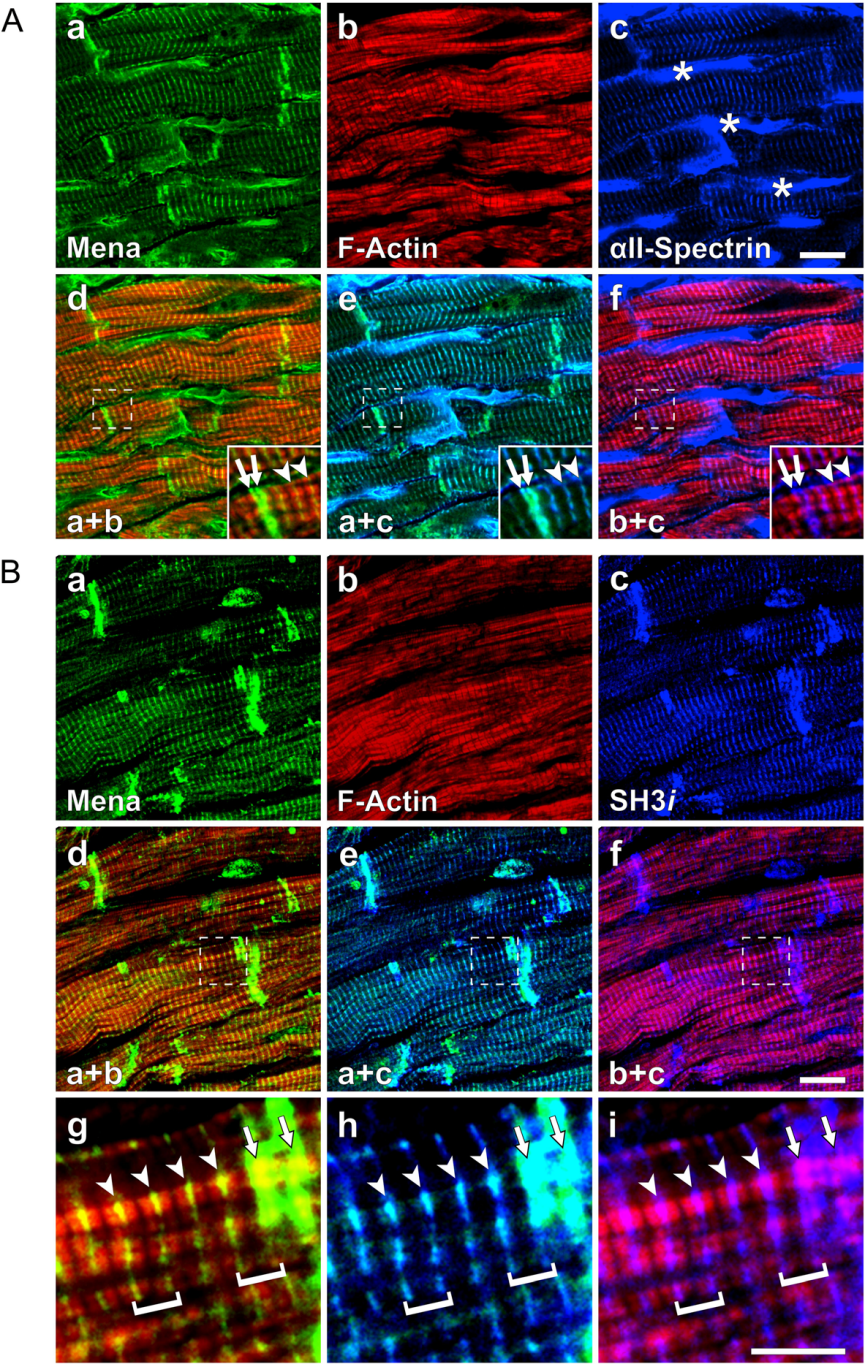
**Mena specifically colocalizes with the αII-Spectrin splice variant SH3*i. *(A**, **B)** Confocal microscopy images of adult mouse heart cryosections stained for Mena (**a**, green), F-actin (**b**, red), and total αII-Spectrin (**A**, image **c**, blue) or the αII-Spectrin splice variant SH3*i* (**B**, image c, blue). **(A)** Mena, F-actin, and a portion of total αII-Spectrin colocalized at Z-discs (**A**, merged images **d**-**f**, arrowheads) and intercalated discs (**A**, merged images **d**-**f**, arrows. Detailed analyses revealed a doublet appearance of the proteins across the intercalated disc (**A**, images **d**-**f**, insets). Most αII-Spectrin was detected at the lateral plasma membrane (**A**, image **c**, asterisks) and colocalization with Mena and F-actin at these sites was minor. **(B)** SH3*i* was exclusively detected at Z- and intercalated discs but was absent from the lateral plasma membrane compare image **c** of **A** and **B**). Merged images **d-f** and magnified views of the indicated areas therein (images **g**-**i**) revealed high degree of colocalization of Mena and SH3*i* at Z-discs **(**images **g**-**i**, arrowheads) and intercalated discs (arrows), the lateral boundaries of the sarcomeres, which are exemplarily highlighted by brackets. Outside Z- and intercalated discs, colocalization of Mena and SH3*i* with F-actin was minor. Scale bars in images **c** and **f** 15 μm, image **i** 5 μm.

αII-Spectrin-deficient mice die from severe cardiac abnormalities that are reminiscent of phenotypes observed following the loss of proteins that regulate actin dynamics, such as Mena and VASP [23]. Thus, the cardiac phenotype observed in αII-Spectrin-deficient mice may, at least in part, be related to the failure to complex with Mena and VASP at Z- and intercalated discs. To test this hypothesis, we compared the sub-cellular distribution of Mena with that of αII-Spectrin and the αII-Spectrin splice variant; SH3*i*, which contains a 20-amino acid insertion C-terminal to the SH3 domain and is also present in the mouse heart [30]. αII-Spectrin was found enriched at the lateral plasma membrane as well as at Z- and intercalated discs, where it colocalized with Mena and F-actin (Figure 9A). More detailed analyses of protein distribution revealed a doublet appearance of Mena and αII-Spectrin across the intercalated disc (Figure 9A) and is consistent with co-localization of the proteins at the transitional junction, a sharp boundary that lies between the last sarcomere and the intercalated disc thin filaments [4,22]. In contrast, the SH3*i* splice variant was exclusively found at Z- and intercalated discs and not at the lateral plasma membrane. Therefore, SH3*i* closely paralleled the subcellular distribution of Mena and indeed the proteins displayed a high degree of colocalization at Z- and intercalated discs (Figure 9B), indicating that SH3*i* may be important for Mena targeting or function in the heart. Consistently, quantitative immunocolocalization analysis [31] revealed significantly higher Pearson’s correlation and Mander’s overlap coefficients of Mena versus SH3*i* than Mena versus total αII-Spectrin (Pearson’s 0.73 versus 0.65; Mander’s 0.94 versus 0.85, n=24, *P<0.05). Importantly, colocalization of Mena and SH3*i* with F-actin was limited to Z- and intercalated discs and no colocalization with sarcomeric F-actin was observed, indicating that the association of Mena and SH3*i* with the α-cardiac actin fibers of the contractile machinery is minor (Figure 9B).

**Figure 10:**
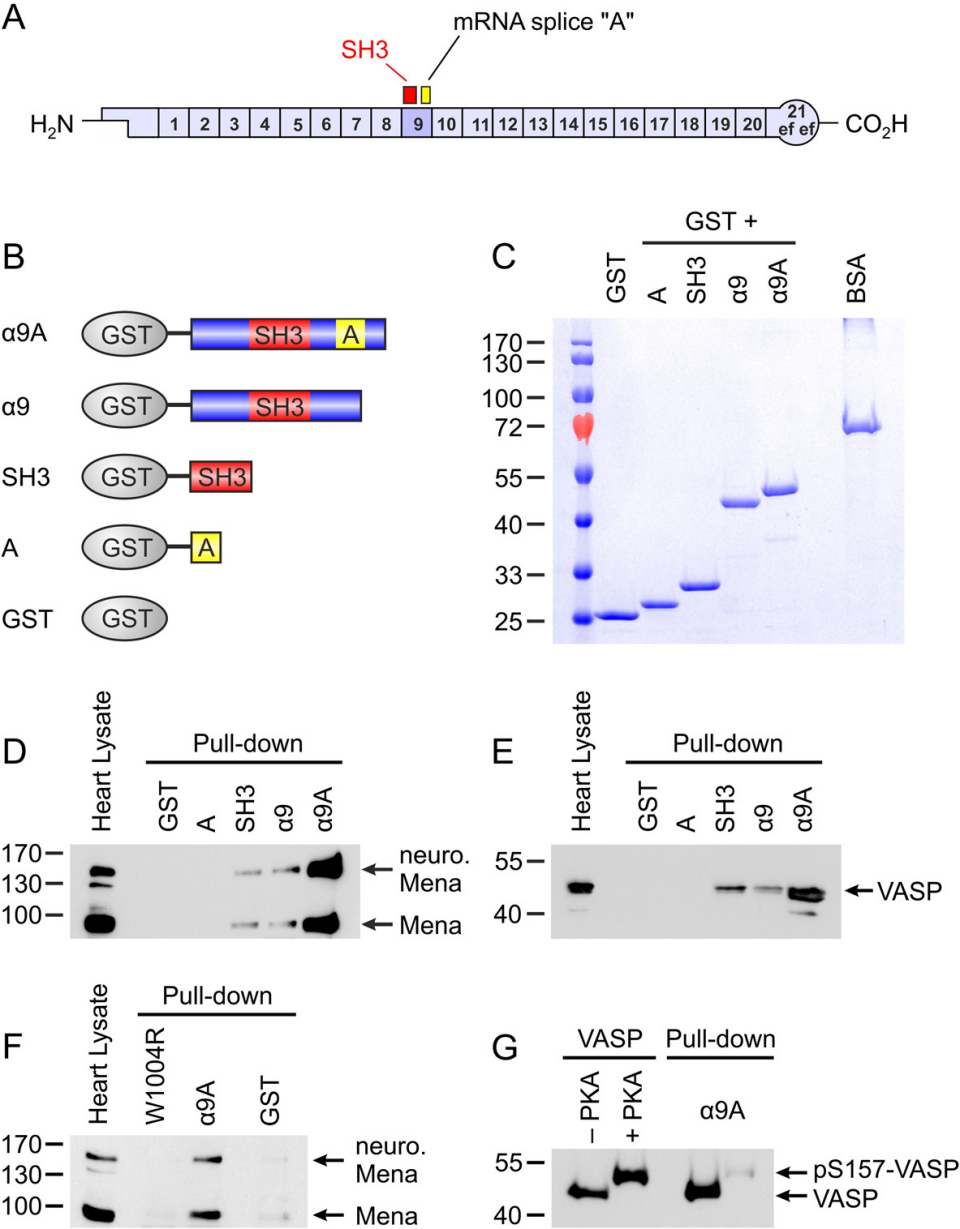
**C-terminal extension of the αII-Spectrin SH3 domain enhances interaction with Mena and VASP. (A)** αII-Spectrin is composed of 21 triple-helical repeats. Two αII-Spectrin splice variants exist in the mouse heart, either with or without a 20-amino acids insertion in the 9^th^ repeat (“A”), C-terminal to the SH3 domain (indicated in red). The splice variant with the insertion is termed SH3*i*. **(B**, **C)** Schematic diagram **(B)** and Coomassie stained gel **(C)** of the purified GST-αII-Spectrin fusion proteins used for the pull-down experiments shown in **D**-**G**. Two microgram bovine serum albumin (BSA) served to calibrate the protein load. **(D**, **E)** Lysates of adult **(D)** and neonatal **(E)** mouse hearts were incubated with the depicted GST-fusion proteins and the precipitated material was blotted with Mena- or VASP-specific antibodies, respectively. **(F)** The interaction between Mena and SH3*i*, requires a functional SH3 domain and mutation of α9A to exchange the conserved Trp1004 residue with an arginine (W1004R) abrogates the binding. **(G)** The interaction between purified recombinant VASP and SH3*i* is regulated by phosphorylation. GST-α9A readily precipitated the non-phosphorylated VASP (46 kDa), but not PKA-phosphorylated protein (pS157-VASP, 50 kDa).

Next, we compared binding of Mena/VASP to general αII-Spectrin and the SH3*i* isoform. The difference between αII-Spectrin and the SH3*i* isoform is a 20 amino acid insertion C-terminal to the SH3 domain in the SH3*i* (Figure 10A). To determine whether or not this insertion could alter αII-Spectrin binding to Mena and VASP, we generated, expressed, and purified a series of GST-fusion proteins (Figure 10B and C). In addition to GST alone, which served as negative control, these GST-fusion proteins included the alternatively spliced 20 amino acids (A), the SH3 domain (SH3), the α9 repeat of αII-Spectrin (α9), and the α9 repeat of SH3*i*, which includes the alternatively spliced 20-amino acids (α9A). In GST pull-down experiments with mouse heart lysate, α9A precipitated at least 10-fold more Mena than α9 or SH3 (Figure 10D). Interestingly, the 20 amino acid insert alone was not sufficient for Mena binding. To analyze VASP binding in the same assay, we performed GST pull-downs with neonatal mouse heart lysates. Similar to Mena, interaction of VASP with α9A was more prominent than the interaction with α9 or SH3 (Figure 10E).

### The interaction between Mena/VASP and SH3*i*, requires a functional SH3 domain and is regulated by phosphorylation

To characterize the formation of the Mena/VASP:α9A complex in more detail we determined whether a functional SH3 domain was essential for binding or whether α9A forms an SH3-independent site for Ena/VASP docking. The binding of SH3 domains to proline-rich ligands is mediated by two conserved hydrophobic pockets within the SH3 domain. To abrogate the binding of the SH3 domain, we substituted a highly conserved tryptophan residue (W1004) which is critically involved in formation of the second hydrophobic binding pocket [32], with an arginine residue to generate the W1004R point mutant. In GST pull-down experiments with mouse heart lysates, Mena binding to W1004R was markedly reduced versus binding to α9A and was comparable to the background binding to GST alone (Figure 10F). We have previously shown that the PKA-mediated phosphorylation of VASP impairs its interaction with the SH3 domain of αII-Spectrin [10]. Because the interaction of Mena and VASP with α9A appeared to be stronger than the interaction with either α9 or SH3 (Figure 10D and E), we investigated whether the binding of α9A to VASP is also regulated by PKA. Therefore, purified recombinant VASP was phosphorylated with activated PKA *in vitro*, which resulted in the complete phosphorylation of the protein on Ser157 (Figure 10G). In subsequent pull-down assays, α9A readily precipitated the non-phosphorylated, but not the PKA-phosphorylated protein (Figure 10G).

Taken together, these data indicate that the αII-Spectrin splice variant SH3*i* is the prominent Mena/VASP interaction partner at Z- and intercalated discs. Moreover, the SH3 domain of SH3*i* is essential for complex formation and PKA-mediated VASP phosphorylation impairs its binding *in vitro*.

### Mena and SH3*i* establish multi-protein complexes at Z- and intercalated discs

**Figure 11:**
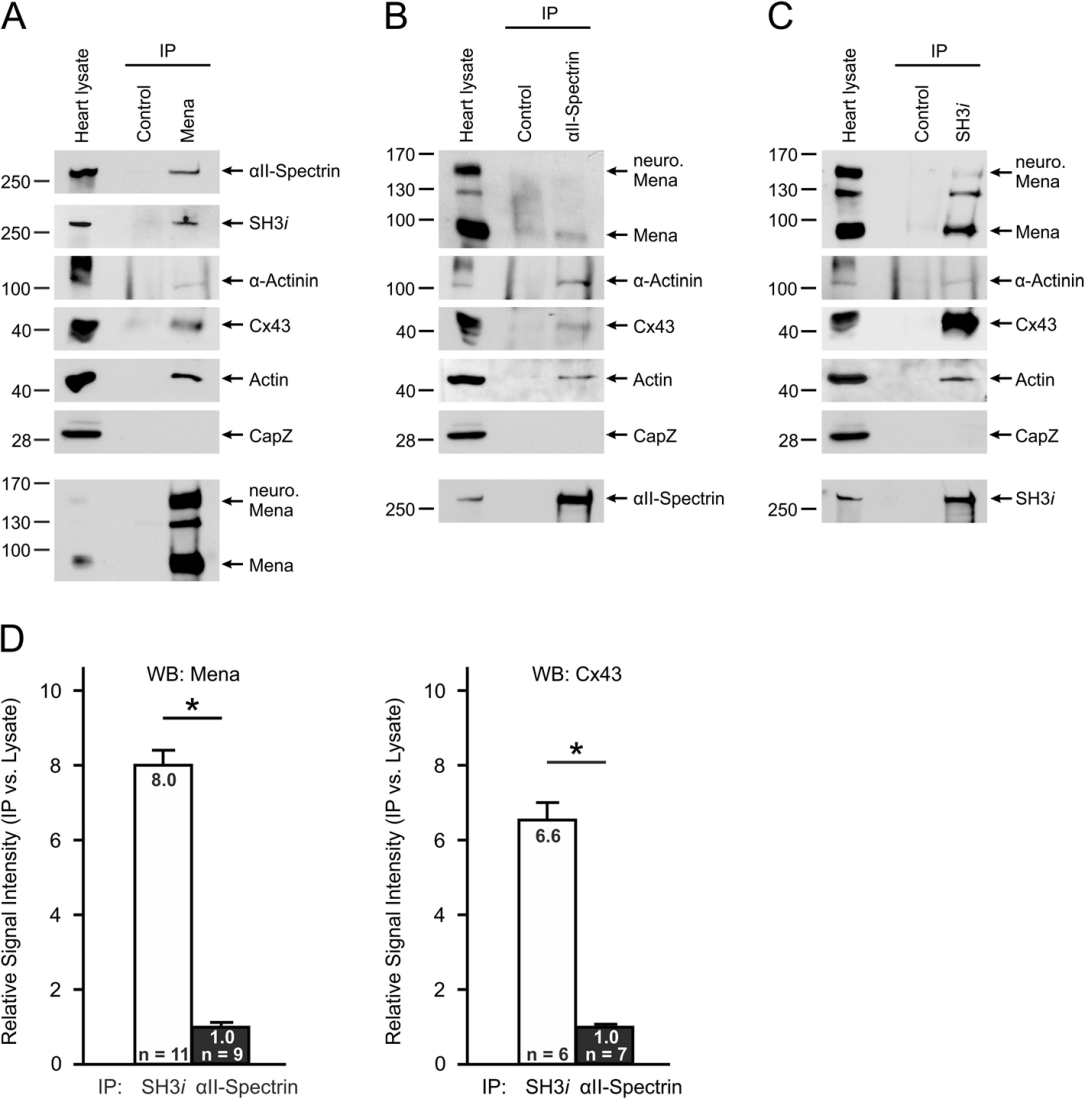
**Mena and SH3*i *establish multi-protein complexes at Z- and intercalated discs.** Representative Western blots of Immunoprecipiation (IP) studies. Lysates of adult mouse hearts were immunoprecipitated with Mena-specific **(A)**, general αII-Spectrin-specific **(B)**, or SH3*i*-specific **(C)** antibodies and the precipitated material was probed with antibodies against total αII-Spectrin, SH3*i*, Mena, actin, the Z-disc components α-Actinin and CapZ, and the intercalated disc marker Connexin 43 (Cx43). Western blot analysis of the precipitated material with the same antibodies that were used for IP served as control **(**lowermost panel in **A**, **B**, and **C)**. Mena, αII-Spectrin, and SH3*i* were found in complexes with actin, α-Actinin and Cx43, but not in complexes with CapZ. **(D)** Statistical analysis of Mena and Cx43 protein amounts in IPs with general αII-Spectrin-specific (black bars) or SH3*i*-specific antibodies (white bars). SH3i precipitated more Mena and Cx43 than αII-Spectrin. Bars represent the mean ± S.E.M (ANOVA, *P<0.05).

To determine whether SH3*i* and Mena form complexes *in vivo* and to elucidate whether or not the proteins are important structural components of Z- and intercalated discs, we performed immunoprecipitation (IP) studies from lysates of adult mouse hearts. Mena-specific antibodies co-precipitated total αII-Spectrin and SH3*i* but the ratio of precipitate to input (heart lysate) was greater for SH3*i* than αII-Spectrin (Figure 11A). Moreover, given that the antibody against αII-Spectrin also detects the SH3*i* splice variant (but not vice versa) it seemed that the preferred interaction is between Mena and the SH3*i* splice variant. Consistently, SH3*i*-specific antibodies precipitated 8-fold more Mena protein than general αII-Spectrin-specific antibodies (Figure 11B-D). Mena was also found to complex with actin, sarcomeric α-actinin, and Cx43 (Figure 11A). We also investigated the interaction of αII-Spectrin or SH3*i* with Cx43 and found that, similar to its binding to Mena, SH3*i* precipitated significantly more Cx43 (6.6-fold) than αII-Spectrin (Figure 11 B-D). In contrast, complex formation of αII-Spectrin or SH3*i* with actin and sarcomeric α-actinin was similar. Ena/VASP proteins associate at or near the barbed ends of actin filaments thereby restricting the access of barbed end capping proteins. In our IP studies, we investigated the association of Mena, αII-Spectrin, and SH3*i* with CapZ, an F-actin capping protein at Z-discs. Although Mena, αII-Spectrin, and SH3*i* are also found at Z-discs in cardiac myocytes, we could not detect a physical association between any of the actin binding proteins and CapZ (Figure 11), indicating a mutually exclusive binding of the proteins to actin.

### Mena/VASP double-deficiency impairs Z- and intercalated disc integrity

**Figure 12:**
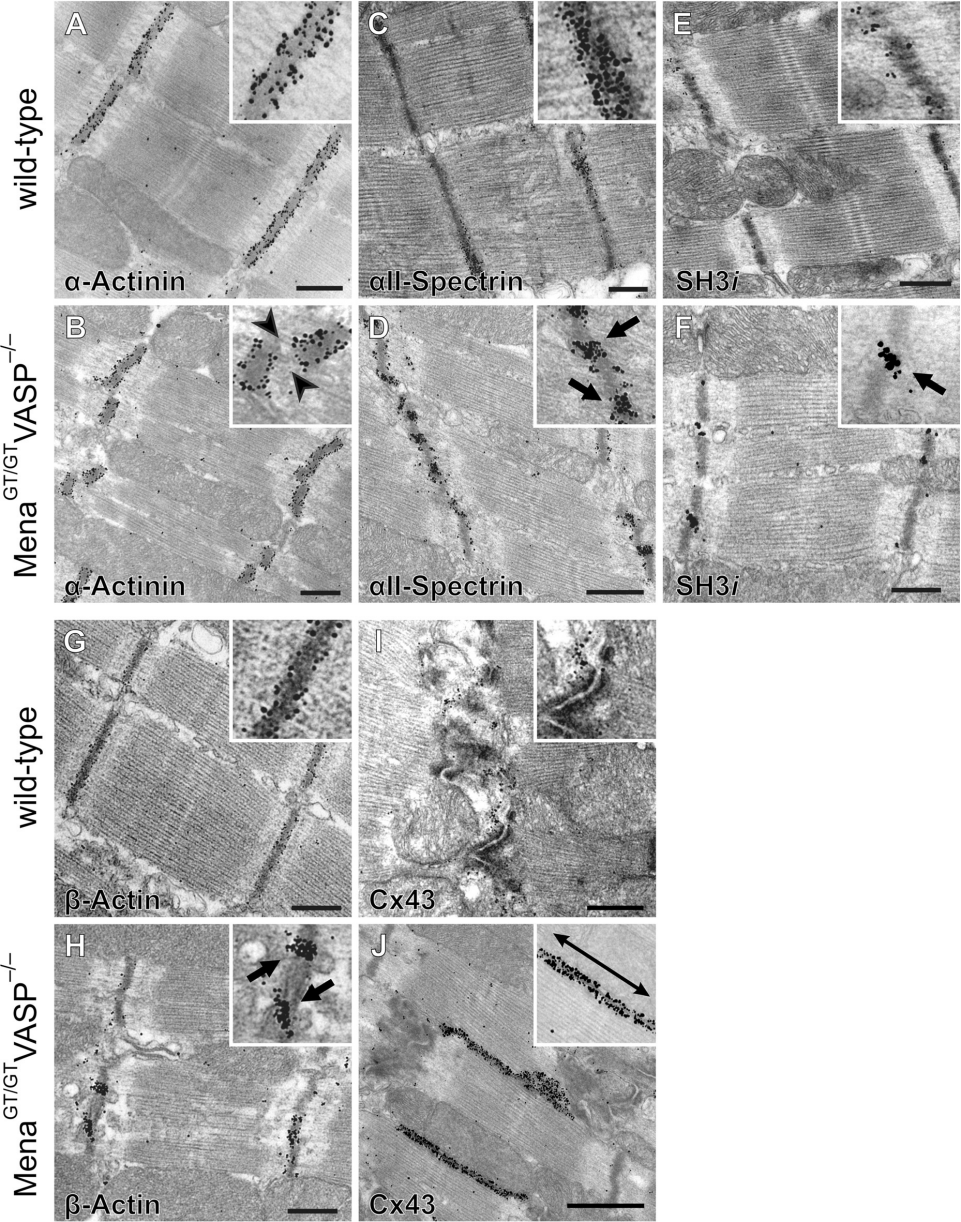
**Mena/VASP double deficiency impairs Z- and intercalated disc integrity.** Immuno-electron microscopy of Z- and intercalated disc regions in wild-type and Mena^GT/GT^VASP^−/−^ mouse papillary muscle. Sections were labelled with antibodies to α-Actinin **(A**, **B)**, αII-Spectrin **(C**, **D)**, SH3*i***(E**, **F)**, β-Actin **(G**, **H)**, and Connexin 43 **(**Cx43, **I**, **J)**. The Z-discs in the Mena^GT/GT^VASP^−/−^ appeared fractured **(B**, arrowheads**)** and clusters of αII-Spectrin, SH3*i*, and β-Actin were found within the ruptured Z-discs and often associated with the emerging gaps (**D**, **F**, **H**; arrows). In contrast to the convoluted organization of intercalated discs in wild-type hearts **(I)**, intercalated discs in Mena^GT/GT^VASP^−/−^ mice displayed a pronounced step-like appearance with elongated and lateralized gap junctions **(J**, double arrow**)**.

Our data revealed that αII-Spectrin/SH3i, Mena, and actin form multi-protein complexes in cardiomyocytes. To test if these complexes are important for the structural integrity of Z- and intercalated discs, we compared the mor-phology of hearts from wild-type and Mena^GT/GT^VASP^−/−^ mice by pre-embedding immunogold electron micro-scopy. First, we focussed on the morphology of the Z-discs and labeled thin sections with anti α-Actinin anti-bodies. In sections from wild-type and Mena^GT/GT^VASP^−/−^ mice, immunogold particles were equally found at Z-discs. However, compared to the wild-type sections, the integrity of the Z-discs in the Mena^GT/GT^VASP^−/−^ mice was markedly altered and demonstrated a wavy and fractured appearance (Figure 12A and B). The thin filaments of the contractile apparatus are anchored at Z-discs and consistent with the ruptured Z-discs in the Mena^GT/GT^VASP^−/−^ hearts, the morphology of actin filaments in the I-band also appeared disorganized and chaotic (Figure 12A and B). Next, we assessed whether Mena/VASP deficiency affects the localization of αII-Spectrin/SH3*i* at Z-discs. In contrast to sections from wild-type hearts where the proteins were almost equally spread along the Z-disc, clusters of αII-Spectrin and SH3*i* were found within the ruptured Z-discs and often associated with the emerging gaps in cardiac Z-discs from Mena^GT/GT^VASP^−/−^ mice (Figure 12C-F). Gamma-actin labeling at the filamentous structures projecting from the Z-disc termini to the mitochondria was indistinguishable in wild-type and Mena^GT/GT^VASP^−/−^ heart sections (data not shown) but the β-actin pattern was markedly altered in Mena^GT/GT^VASP^−/−^ hearts. Similar to the αII-Spectrin/SH3*i* labeling, β-actin immunogold particles clustered in the fractured Z-discs (Figure 12G and H).

Finally, we compared the morphology of intercalated discs in wild-type and Mena^GT/GT^VASP^−/−^ mice and analyzed Connexin 43 localization. Whereas cardiac muscle from wild-type mice displayed the typical convoluted organization of intercalated discs with compact gap junctions intimately connected with adjacent desmosomes and adherens junctions, intercalated discs in Mena^GT/GT^VASP^−/−^ mice displayed a pronounced step-like appearance with elongated, lateralized gap junctions. Indeed, adherens junctions at both sides of the elongated gap junctions were separated by almost the entire length of a sarcomere (Figure 12I and J).

Thus, Mena/VASP double-deficiency results in structural alteration of Z- and intercalated discs. This may explain the conduction delays and the development of the dilated cardiomyopathy in Mena^GT/GT^VASP^−/−^ mice and supports the hypothesis of an important role of β-cytoplasmic actin networks for the mechanical support, myofibrillogenesis, and ion channel function in the heart.

## Discussion

In the present study, we analyzed the physiological functions of Mena and VASP in the mammalian heart and investigated the role of the proteins in the organization of cytoplasmic actin networks.

### Stage-dependent expression of cytoskeletal proteins in the mouse heart

We found that cardiac VASP expression was upregulated in lysates from 1-week-old and adult hypertrophic hearts, conditions in which the heart is confronted with a sudden and dramatic increase in mechanical workload. Mena expression followed a similar trend, but the protein is more robustly expressed in the adult heart, pointing to a more specialized role of VASP in the developing or remodelling heart. The upregulation of the proteins in the infant and hypertrophied heart could partially be due to non-cardiomyocyte cells and fibrosis. However, Mena and VASP protein levels were also upregulated in cardiac myocytes isolated from mice subjected to ascending aortic constriction [33] and from the developing rat heart [7], demonstrating that the increased protein expression is likely cardiomyocyte-specific. Consistent with activation of β-adrenergic signaling pathways, VASP from infant and hypertrophied hearts was frequently PKA phosphorylated and displayed its characteristic doublet on Western blots. How PKA-mediated phosphorylation regulates VASP function under these conditions is currently unknown and requires further investigations.

Taken together, Mena and VASP function may be especially important in heart development and remodeling. However, additional studies on the role of the proteins in embryonic and postnatal development and in hypertrophic conditions, including transverse aortic constriction or sustained activation of β-adrenergic signaling, are required to experimentally address this hypothesis.

### Cardiac phenotype of Mena/VASP single- and double-deficient mice

The present study is the first to systematically address the consequences of Mena-, VASP-, and Mena/VASP double-deficiency in cardiac physiology.

Together with the histology data, our echocardiographic and hemodynamic measurements revealed an important role of Mena and VASP for the mechanical properties of the heart. Left-ventricular performance was increasingly impaired in adult VASP-, Mena-, and Mena/VASP double-deficient mice and only the double-deficient animals developed dilated cardiomyopathy. Consistent with a higher expression level of Mena in the adult heart, this suggests that Mena is more important for the mechanical properties of the heart than VASP. Because conventional Mena^−/−^ mice display a cardiac phenotype, which is comparable to our Mena^GT/GT^ mice [34], the remaining Mena expression in the brain of our Mena^GT/GT^ mice does not seem to influence cardiac performance. With respect to cardiac mechanics, Mena and VASP can, to a certain extent, compensate for the loss of the other family member. However, the functional compensation is not sufficient to preserve full cardiac performance in the single gene-deficient animals. We investigated Mena protein levels in VASP-deficient animals and vice versa. However, consistent with our previous findings [16], we could not detect a compensatory upregulation of the proteins (data not shown).

When we investigated the electrical conduction in wild-type and mutant hearts, intra-atrial and intra-ventricular propagation of electrical signals was equally delayed in Mena/VASP single- and double-deficient mice. In contrast to heart mechanics, this suggests that Mena and VASP have indispensable functions in the propagation of electrical signals, likely in the formation of the gap junctions in intercalated discs.

Previously, a dominant negative strategy with cardiac-specific overexpression of the VASP-EVH1 domain was used to disrupt the *in vivo* function of Mena and VASP [35]. Animals with high transgene expression developed cardiac abnormalities and died within 2–6 weeks after birth [35]. Because our Mena/VASP double-deficient mice did not reveal an obviously reduced lifespan, it is likely that the transgene caused off-target effects and possibly affected other EVH1 domain-containing proteins.

### Mena/VASP, SH3*i*, and actin assemble multi-protein complexes at Z- and intercalated discs

Our results revealed that Mena/VASP specifically interact with a distinct αII-Spectrin splice variant (SH3*i*), which is exclusively localized at Z- and intercalated discs of cardiomyocytes. The SH3*i* isoform contains a 20 amino acid insertion C-terminal to the SH3 domain, which enhances the binding to Mena and VASP in pull-down assays. Similar to the isolated SH3 domain [10], PKA-mediated VASP phosphorylation abrogates binding of SH3*i* to VASP *in vitro*. However, it remains to be detailed whether activation of β-adrenergic signaling pathways affects the Mena/VASP: SH3*i* complex formation *in vivo*. Our immunoprecipitation experiments suggested that Mena, SH3*i,* and actin assemble multi-protein complexes at Z- and intercalated discs. Interestingly, we could not detect other actin-binding proteins such as CapZ in these complexes, arguing for the existence of distinct actin networks in cardiomyocytes. We used immunogold labeling to determine the distribution of Mena in cardiac myocytes. At Z- and intercalated discs of mouse cardiomyocytes, Mena localized to the edges of the sarcomeres, where the thin filaments are anchored. Importantly, we could show for the first time that actin filaments at these sites are, at least in part, composed of the β-cytoplasmic actin isoform. In contrast, colocalization of Mena with the thin filaments of the contractile machinery or the filamentous structures projecting from the Z-disc termini towards the mitochondria was minor. This suggests that in the adult mouse heart Mena is not involved in the regulation of α-cardiac or γ-cytoplasmic actin fibers but specifically interacts with β-cytoplasmic actin networks. However, currently it is unknown how Mena and VASP may distinguish between different actin isoforms and two commonly held concepts may give an explanation. First, Mena and VASP could discriminate between muscle and cytoplasmic actin isoforms, which has been shown for a subset of other actin-binding proteins including Thymosin b4 and profilin [1]. All actin isoforms share at least 93% identity and β- and γ-actin differ by only four residues at the N-terminus. Up to now, the actin epitope, which binds to Mena or VASP, has not been identified. It is tempting to speculate that Mena and VASP may favor binding to cytoplasmic actin isoforms. Indeed most cellular effects of Mena and VASP, such as endothelial barrier function, fibroblast migration, and axon guidance, are associated with the regulation of β-actin actin dynamics [10,11,36]. Moreover, profilin, which directly interacts with Mena and VASP and increases the anti-capping and filament elongation activities of the proteins, binds cytoplasmic actin with higher affinity than muscle actin [37]. Nevertheless, there may be a second explanation for the isoform-specific interaction of the proteins with actin. Mena and VASP may be localized to distinct subcellular regions, perhaps as a result of differential interactions with other proteins such as αII-Spectrin. Because these interactions can be regulated by cyclic nucleotide-dependent signaling pathways, this may allow the cell to modulate actin dynamics in response to external stimuli in a spatial and temporal defined manner. In agreement with this concept, VASP readily increases the filament assembly of *muscle* actin *in vitro*[9,11], arguing against an isoform-selective actin binding of VASP per se. To our knowledge, this is the first study indicating that Mena and VASP interact with actin fibers in an isoform-dependent manner.

### Mena/VASP double-deficiency impairs Z- and intercalated disc integrity

When we analyzed the morphology of cardiomyocytes in our Mena^GT/GT^VASP^−/−^ mice, Z-discs appeared wavy and fractured. Furthermore αII-Spectrin, SH3*i*, and β-actin formed clusters within the Z-discs and actin filaments of the I-band appeared disorganized. It is tempting to speculate that Mena/VASP deficiency results in a disruption of the multi-protein complexes with αII-Spectrin and β-actin and thus impairs the structural integrity of the Z-discs, which in turn disrupts the anchoring of the thin filaments from the I-band. However, we cannot rule out that the ruptured Z-discs are the primary consequence of Mena/VASP double-deficiency and that clustering of αII-Spectrin and β-actin are secondary effects. One finding that argues against a secondary effect of αII-Spectrin and β-actin clustering, however, is the sub-cellular distribution of α-Actinin in wild-type and Mena^GT/GT^VASP^−/−^ mice. Although the Z-disc structure was clearly impaired in the Mena^GT/GT^VASP^−/−^ mice, the α-Actinin staining per se was indistinguishable from wild-type mice, indicating that in contrast to αII-Spectrin and β-actin other components of the actin network at Z-discs are not affected by the malformation of the Z-discs.

Mena/VASP double-deficiency also impaired the structural integrity of intercalated discs. In our Mena^GT/GT^VASP^−/−^ mice, especially the morphology of gap junctions was markedly changed. In the double-deficient animals, Connexin 43-positive gap junctions were excessively lateralized and elongated and clearly separated from desmosomes and adherens junctions. Currently, information how actin networks affect gap junction formation is sparse. Interactions of connexins with the actin cytoskeleton and associated proteins appear to stabilize gap junctions at the plasma membrane and αII-Spectrin has been shown to co-precipitate with Cx43, likely via interaction with ZO-1 [38]. Our data indicate that especially SH3*i* may be involved in cytoskeletal organization at gap junctions as SH3*i* precipitated significantly more Cx43 and Mena than the general αII-Spectrin splice variant. In a complex with Cx43, ZO-1, and SH3*i*, Mena (and VASP) may regulate cytoplasmic networks and thereby stabilize the assembly and intercellular communication of gap junctions. In agreement with this hypothesis, blocking of actin dynamics by cytochalasin B treatment or blocking the association of Cx43 with the actin cytoskeleton led to a significant increase in the gap junction size [39,40]. Nevertheless, it remains unclear, why the enlarged gap junctions in our Mena^GT/GT^VASP^−/−^ mice result in an impaired electrical conduction and prolonged PQ and QRS complexes in ECG recordings. Possibly, because of the lateralization of the gap junctions or a defective formation of gap junction channels between opposing cells. However, further experiments are required to address this question.

## Conclusions

**Figure 13:**
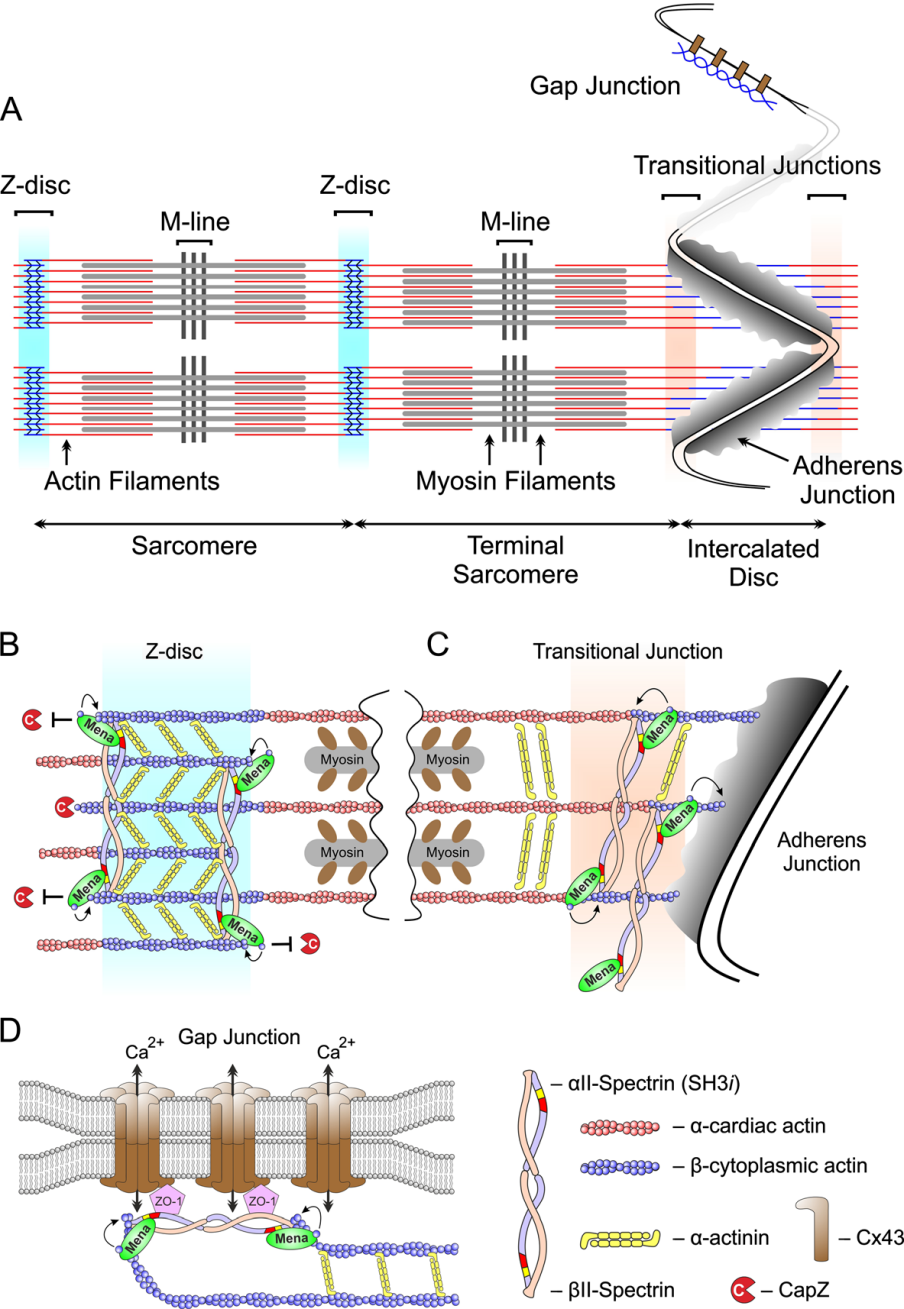
**Proposed role of Mena/VASP and αII-Spectrin for cytoplasmic actin assembly in the mammalian heart. (A)** Diagram of myofibrils and junctions in two adjacent cardiomyocytes. The longitudinal force generated by sliding of actin and myosin fibers along each other is transduced between adjacent sarcomeres at the Z-disc and between adjacent cardiomyocytes at the adherens junctions of the intercalated disc. In contrast to the transverse adherens junctions, which are located at the end of myofibrils, gap junctions are often found in close association with mitochondria and and propagate electrical signals between cells by exchange of calcium and sodium ions. Whereas actin fibers of the contractile machinery are mainly composed of α-cardiac actin (red), actin filaments at gap junctions and the edges of Z- and intercalated discs, where the thin filaments are anchored, are composed of β-cytoplasmic actin (blue). **(B**-**D)** Proposed mechanism how Mena/VASP and αII-Spectrin are thought to regulate cytoplasmic actin networks at Z-discs **(B)**, intercalated discs **(C)**, and gap junctions **(D)**. For simplicity reasons, only Mena is depicted. Complexes of Mena/VASP and SH3*i* regulate β-actin networks by association at or near the barbed ends of β-actin filaments. Possibly, these complexes protect the barbed ends of thin filaments from capping by CapZ or modulate the geometry of the actin network by the intrinsic actin polymerization/bundling activity of Mena and VASP. Notably, heterotetrameric Spectrins, which are composed of two α- and two β-subunits, also regulate actin networks and are well known F-actin crosslinking proteins. Therefore disrupted Mena/VASP:SH3*i* complex formation in Mena^GT/GT^VASP^−/−^ mice may explain the disturbed morphology of Z- and intercalated discs, and the development of dilated cardiomyopathy and conduction abnormalities.

We propose a model how Mena/VASP and αII-Spectrin regulate β-cytoplasmic actin networks at Z- and intercalated discs of the mammalian heart (Figure 13). Complexes of Mena/VASP and SH3*i* regulate β-actin networks by association at or near the barbed ends of β-actin filaments. Possibly, these complexes protect the barbed ends of thin filaments from capping or modulate the geometry of the actin network with implications for mechanical support, myofibrilogenesis, and ion channel function. Conversely, disrupted Mena/SH3*i* complex formation in Mena^GT/GT^VASP^−/−^ mice impairs the integrity of Z- and intercalated discs and results in dilated cardiomyopathy and conduction abnormalities.

## Methods

### Generation of Mena^GT/GT^ and Mena^GT/GT^VASP^−/−^ mice

The mouse Mena gene is located on chromosome 1 spanning about 115 kb of genomic sequence (BLAST-like alignment tool, BLAT [41], Assembly NCBI37/mm9). To disrupt the Mena gene in mice, we generated a mouse strain, using the RRG138 embryonic stem (ES) cell line from BayGenomics (http://www.genetrap.org). In RRG138, the gene trap vector pGT0Lxf is inserted between exons 2 and 3 of the mouse Mena gene (Figure 2) and single insertion of the vector was confirmed by Southern blot analysis. 129/Ola ES cells heterozygous for the targeted mutation were microinjected into C57Bl/6 blastocysts and implanted into pseudopregnant foster mothers. Male chimeras were mated with C57Bl/6 females to obtain heterozygous Mena^+/GT^ mice. Offspring were genotyped using a forward primer located in exon 2 of the mouse Mena gene (me_F3: 5′ – TGGGCAGAAAGATTCAAGACC – 3′) either in combination with a reverse primer in Mena intron 2 (me_in2_R2: 5′ – CCAGTTTCAATGCCCATTCCTT – 3′) to amplify the 1123 bp wild-type fragment or in combination with a reverse primer in the gene trap vector (pGT0Lxf_R2: 5′ – CGGATCTCAAACTCTCCTCC – 3′) to amplify the 824 bp knockout fragment. TA-cloning and sequencing of the knockout PCR product confirmed targeting of the mouse Mena gene and allowed to map the insertion of the gene trap vector 682 bp downstream of Mena exon 2. VASP-deficient mice have been described previously [16] and were mated to Mena^GT/GT^ mice to generate Mena/VASP double-deficient animals (Mena^GT/GT^VASP^−/−^). In the present study, 8–12 months old VASP-, Mena-, and Mena/VASP double-deficient mice and controls of a mixed 129/Ola X C57Bl/6 background were used for all experiments.

### Animal experiments

All animal experiments were performed according to the relevant regulatory standards and were approved by the local government (license to KS and PMB, #621-2531.01-25/05, #55.2-2531.01-62/08, and #55.2-2531.01-95/11).

#### Transverse aortic constriction (TAC)

Male mice were anesthetized using isoflurane and the transversal aorta was prepared after jugular incision. A 27-gauge needle was tied against the aorta using a 5–0 non-absorbable suture. After removal of the 27-gauge needle, skin was closed, and the mice were kept on a warming plate until recovery from anesthesia. After 3 weeks of TAC, mice were then sacrificed under anaesthesia, the hearts were weighed and the left ventricles were dissected for protein extraction.

#### Echocardiography

Serial transthoracic echocardiography (Aplio, Toshiba, Netherlands) was performed under light isoflurane anaesthesia and spontaneous respiration by a single researcher experienced in rodent echocardiography blin-ded to mouse genotype. From two-dimensional short axis imaging, endocardial borders were traced at end-systole and end-diastole utilizing a prototype off-line analysis system (NICE, Toshiba Medical Systems, The Netherlands). Measurements were performed at the mid-papillary muscle level. The end-systolic (smallest) and enddiastolic (largest) cavity areas were determined. Using the end-systolic (ESA) and-diastolic areas (EDA), fractional area changes were calculated ((EDA-ESA)/EDA). Only animals with a heart rate greater than 450/min were included.

#### Invasive hemodynamics

Terminal measurements of left ventricular pressure (LVP) were performed in 1% isoflurane anesthetized mice with a 1.4F micromanometer-tipped catheter and MPVS Ultra amplifier (Millar Instruments). After 30 min of stabilization, 30 sec of data were collected and analyzed with Chart 7.3 from ADInstruments running on PowerLab 16/30, including heart rate, systolic and diastolic LVP, and the first derivatives of LVP, ±dP/dt. Only animals with a heart rate greater than 450/min were included.

#### High resolution ECG recordings

Mice were anesthetized using isoflurane (0.8%) and kept on a warming plate (37°C) throughout the experiment. Lead I ECG recordings were captured at a sampling rate of 4000 points per second (ADInstruments, PowerLab 8/35 with BIO amplifier) and original ECG recordings were analyzed with the ecg-auto software (version 2.5.0.3, emka technologies).

### Antibodies and fluorescent phalloidin

Polyclonal rabbit anti-Mena antibodies were raised against a purified glutathione-S-transferase (GST, GE-Healthcare) fusion protein comprising mouse Mena amino acids L147-L279 (numbering according to NCBI protein ID AAC52863). Antibodies were immunoselected with a purified maltose-binding fusion protein (MBP, New England Biolabs) comprising the same mouse Mena residues. For double-immunofluorescence studies, we conjugated the purified Mena antibodies with fluorescein isothiocyanate (FITC, Sigma). Rabbit antibodies against the general αII-Spectrin protein isoform were generated and immunoselected with a purified GST fusion protein comprising amino acids E970-D1025 of the human αII-Spectrin sequence (protein ID AAH53521). To remove αI-Spectrin- and GST-specific antibodies, the purified antiserum was immunodepleted with an excess of a GST fusion protein comprising αI-Spectrin residues A978-A1033 (protein ID NP_035595). Other antibodies used were anti-VASP (IG-731, Immunoglobe), anti-SH3*i*[30], anti-GST [10], anti-glyceraldehyde-3-phosphate dehydrogenase (GAPDH, mAB 374, Chemicon), anti-β-Tubulin (T8328, Sigma), anti-β-cytoplasmic actin (13E5, Cell Signalling; A1978, Sigma), anti-γ-cytoplasmic actin (A8481, Sigma), anti-CapZ (AB6017, Millipore), anti-Cx43 (sc-6560, Santa Cruz; 710700, Invitrogen), anti-α-Actinin (A7811, Sigma). Horseradish peroxidase-conjugated secondary antibodies were from Dianova or eBioscience, fluorescence-conjugated antibodies (Alexa-fluor 488, 594, or 647) from Molecular Probes. Fluorescent phalloidin (P1951, Sigma; A22287, Invitrogen) was used to visualize F-actin.

### Organ lysates and Western blotting

For Western blotting, 100 mg mouse organ was homogenized in 2 ml lysis buffer (2% SDS, 1x PBS, 2x complete EDTA-free protease inhibitors (Roche)) using an Ultra Turrax. Following centrifugation for 30 min at 20,000 x g, protein content of the supernatant was determined by UV spectroscopy and 30 μg total protein was resolved by polyacrylamide gel electrophoresis and transferred to nitrocellulose sheets. Membranes were blocked in 5% skimmed milk in PBS, incubated with primary and horseradish peroxidase-conjugated secondary antibodies in blocking solution, and detection was performed with a chemiluminescence substrate (ECL Plus, GE-Healthcare). Equal protein loading was confirmed with β-Tubulin- or GAPDH-specific antibodies.

### Histological analyses

For histological analysis, hearts were fixed in a neutral buffered methanol-free formalin solution for 24 h and then embedded in paraffin. 5 μm transverse sections from the middle of wild-type and Mena/VASP double-deficient hearts were stained with hematoxilin/eosin or 0.1% picrosirius red according to standard protocols. Representative photographs of the transilluminated stained sections on a Zeiss stage micrometer were collected with a Canon EOS 1000D digital camera.

For the analysis of cross sectional cell sizes, standard HE-stained heart sections were mounted with VectaShield mounting medium (Vector Laboratories), images were taken with a Keyence BZ digital microscope (Keyence, Germany), and cross-sectional areas of myocytes displaying round-shaped nuclei were quantified using the ImageJ software (NIH).

### RNA preparation and quantitative real-time PCR analysis

RNA was extracted from frozen LV samples of 6 male mice per group using a Qiagen tissue RNA kit. cDNA was synthesized from 100 ng of total RNA with Superscript (Invitrogen). Quantitative real-time PCR was performed (iCycler, Biorad) with commercially available TaqMan probes for ANP, β-MHC, SMA, Col I, Col III, and GAPDH (Invitrogen). Target gene mRNA levels in Mena^GT/GT^VASP^−/−^ mice were normalized to GAPDH expressed relative to wild-type mice using the 2^(-ΔΔCT) method.

### Isolation of cardiomyocytes

Mice were anesthetized in a gas chamber with isoflurane, hearts were excised and mounted on a Langendorff perfusion-system and were perfused with a nominally calcium-free Tyrodes’ solution containing (in mM) NaCl 113, KCl 4.7, KH_2_PO_4_ 0.6, Na_2_HPO_4_x2H_2_O 0.6, MgSO_4_x7H_2_O 1.2, NaHCO_3_ 12, KHCO_3_ 10, HEPES 10, Taurine 30, BDM 10, glucose 5.5, phenol-red 0.032 for 4 min at 37°C and pH 7.4. Then, perfusion solution was switched to the same solution containing 7.5 mg/ml liberase 1 (Roche diagnostics, Mannheim, Germany), trypsin 0.6% and 0.125 mM CaCl_2_. Perfusion was continued for about 3 min until the heart became flaccid. Ventricular tissue was removed, cut into small pieces and dispersed until no solid cardiac tissue was left. Shortly after, cells were on plated Laminin-coated chamber slides (Nunc) and then prepared for immunofluorescence microscopy.

### GST pull-down and immunoprecipitation experiments

cDNA parts of the general mouse αII-Spectrin (protein ID CAM46235) or SH3*i* (AAI50942) isoform were subcloned into pGEX-6P2 vector (GE Healthcare) to generate constructs termed “A”, “SH3”, “α9”, and “α9A” (compare Figure 10A and B). Except for the alternatively spliced 20 amino acids TRITKEAGSVSLRMKQVEEL, which are only present in SH3*i*, the two protein isoforms are identical. A and α9A are specific for SH3*i* and encode amino acids T1053-L1072 and Q893-E1088, respectively. The W1004R mutant was obtained from α9A by replacing Trp1004 with an arginine residue by site-directed mutagenesis. α9 is specific for the general αII-Spectrin isoform and encodes amino acids Q893-E1068. GST+SH3 encodes the SH3 domain, which is present in both isoforms (E970-D1025). GST and the four GST-fusion proteins were expressed in *E.coli* and affinity purified on glutathione sepharose beads (compare Figure 10C). For GST pull-down assays, hearts of wild-type mice (100 mg) were homogenized in ice-cold 40 mM Hepes-NaOH, pH 7.4, 75 mM NaCl, 1% NP40, and protease inhibitors (Roche) using a Polytron PT3100 homogenizer equipped with a PT-DA 3007 dispersing aggregate. Lysates were cleared by centrifugation (17,000 x g, 4°C, 15 min) and incubated with 5 μg immobilized GST or equimolar amounts of the GST-fusion proteins. Precipitated material was analyzed with anti-Mena and anti-VASP antibodies. GST pull-down assays with purified recombinant VASP were performed essentially as described [10].

For immunoprecipitation experiments, heart lysates were prepared as described above and then incubated with 5 μg purified anti-Mena, anti-αII-Spectrin, or anti-SH3*i* antibodies, followed by protein A-sepharose (GE Healthcare). Purified GST-specific antibodies served as control. After extensive washing in lysis buffer, precipitates were resuspended in SDS sample buffer, resolved by SDS-PAGE, and analyzed by Western blotting employing standard methods. To quantify the amount of Mena and Cx43, which is co-precipitated with the general αII-Spectrin or the SH3*i* variant, respectively, we performed densitometric scans (ImageJ version 1.45s, National Institute of Health, USA). For each IP/Western blot, the signal ratio of precipitated protein vs. a constant amount of heart lysate was calculated. For Mena and Cx43, ratios of SH3*i* precipitations are given relative to the ratios of αII-Spectrin precipitations.

### Immunohistochemistry

All incubation and washing steps were performed at room temperature in a buffer containing 10 mM PIPES pH6.8, 150 mM NaCl, 5 mM EGTA, 5 mM glucose, 5 mM MgCl_2_. 7 μm mouse heart cryosections or isolated mouse cardiomyocytes were fixed in 4% paraformaldehyde, extensively washed with 50 mM ammonium chloride, permeabilized with 0.1% Triton, blocked in 10% goat serum for 1 h, and overnight incubated with primary antibodies in 10% goat serum. Following extensive washing, samples were incubated with the fluorescence-conjugated secondary antibodies and fluorescence-conjugated phalloidin for 1.5 h. After additional three rounds of washing, samples were incubated with FITC-conjugated Mena antibodies, washed again, and mounted with Mowiol (Hoechst). Stained sections were investigated using a Leica SP5 confocal microscope equipped with a 100-fold oil immersion objective. Images were acquired and prepared for presentation using the LAS AF 2.6.0 software from Leica. Pearson’s correlation and Mander’s overlap coefficients were calculated using the Intensity Correlation Analysis Plugin available for ImageJ.

### Pre-embedding Immuno-electron microscopy

Mouse cardiac tissue used for this study was fixed in 4% paraformaldehyde, 15% saturated picric acid in 0.1 M phosphate buffer, pH 7.4, overnight at 4°C. Longitudinal sections were cut on a vibratome (Leica VT 1000S) at a thickness of 50 μm, blocked in 20% NGS in PBS and were incubated with primary antibodies in phosphate-buffered saline containing 5% normal goat serum (Vector Laboratories, Burlingame, CA, USA) overnight at 4°C. After washes in PBS, the sections were incubated with 1.4 nm gold-coupled secondary antibodies in PBS (Nanoprobes, Stony Brook, NY, USA) overnight at 4°C. After several washes the sections were postfixed in 1% glutaraldehyde in PBS for 10 min and then reacted with HQ Silver kit (Nanoprobes). After treatment with OsO_4_, sections were stained with uranyl acetate, dehydrated, and embedded in Durcupan resin (Fluka, Switzerland). Ultrathin sections were prepared (Ultracut S; Leica, Germany) and examined with a ZEISS 910 electron microscope.

## Abbreviations

Cx43: Connexin 43; DCM: Dilated cardiomyopathy; dKO: Mena/VASP double-deficient; ES: Embryonic stem; EVH: Ena/VASP homology; GST: Glutathione-S-transferase; GT: Gene trap; IP: Immunoprecipitation; Mena: Mammalian enabled; M-KO: Mena-deficient; PKA: cAMP-dependent protein kinase; PKG: cGMP-dependent protein kinase; PRR: Proline-rich region; SH3: Src homology 3; SH3i: αII-Spectrin splice variant; VASP: Vasodilator-stimulated phosphoprotein; V-KO: VASP-deficient.

## Competing interests

The authors declare that they have no competing interests.

## Authors’ contributions

PMB, SF, and KS conceived and designed the study; PMB, CJM, KO, MA, BB, HW, SG, SMF, and AU performed the experiments; TF, JAU, IMA, SMF, TR, and SF contributed reagents and materials; PMB, CJM, MA, BB, SF, AU, MU, and KS analyzed the data; PMB and KS wrote the paper; SMF, TR, IF, WL, and SF critically reviewed the paper for important intellectual content. All authors read and approved the manuscript.
